# Cell surface nucleolin is a novel ADAMTS5 receptor mediating endothelial cell apoptosis

**DOI:** 10.1038/s41419-022-04618-x

**Published:** 2022-02-23

**Authors:** Dogan Can Kirman, Bhuvanasundar Renganathan, Wai Kit Chui, Ming Wei Chen, Neslihan Arife Kaya, Ruowen Ge

**Affiliations:** 1 Department of Biological Sciences, Faculty of Science, National University of Singapore, Singapore, 117543 Singapore; 2 School of Biological Sciences, Nanyang Technological University, Singapore, 637551 Singapore; 3 Genome Institute of Singapore, Agency for Science, Technology and Research, Singapore, 138672 Singapore

**Keywords:** Tumour angiogenesis, Apoptosis, Protein translocation

## Abstract

A Disintegrin and Metalloproteinase with ThromboSpondin motif (ADAMTS) 5 functions as an anti-angiogenic and anti-cancer protein independent of its metalloproteinase activity. Both full-length ADAMTS5 and TS5-p45, the autocatalytically cleaved C-terminal 45 kDa truncate of ADAMTS5, inhibits angiogenesis, and induces endothelial cell (EC) apoptosis. However, how ADAMTS5 triggers EC apoptosis remains unclear. This work shows that caspase-8 (Cas-8) and caspase-9 (Cas-9) are involved in TS5-p45-induced EC apoptosis. We identify cell surface nucleolin (NCL) as a novel high-affinity receptor for TS5-p45 in ECs, mediating TS5-p45’s cell surface binding and pro-apoptotic function. We show that the central RNA-binding domain (RBD) of NCL is essential and sufficient for its binding to TS5-p45. Upon interacting with EC surface NCL, TS5-p45 is internalized through clathrin- and caveolin-dependent endocytosis and trafficked to the nucleus via late endosomes (LEs). We demonstrate that the nuclear trafficking of TS5-p45 is important for its pro-apoptotic activity as disruption of LE membrane integrity with an endosomolytic peptide suppressed both nuclear trafficking and pro-apoptotic activity of TS5-p45. Through cell surface biotinylation, we revealed that cell surface NCL shuttles extracellular TS5-p45 to the nucleus to mediate apoptosis. Furthermore, blocking the importin α1/ß1 receptor hindered the nuclear trafficking of TS5-p45, suggesting the involvement of the nuclear importing machinery for this nuclear translocation. RNA-seq identified many apoptosis-related genes that are differentially expressed at least two-fold in TS5-p45-treated ECs, with 10 of them qRT-PCR-validated and at least 5 of these genes potentially contributing to TS5-p45-NCL-induced apoptosis. Altogether, our work identifies NCL as a novel cell surface receptor for ADAMTS5 and demonstrates the critical role of NCL-mediated internalization and nuclear trafficking for ADAMTS5-induced EC apoptosis. These findings reveal novel mechanistic insights of the secreted metalloproteinase ADAMTS5 in angiogenesis inhibition.

## Introduction

Angiogenesis, the formation of new blood vessels from the existing vasculature, is one of the hallmarks of cancer. It is essential for tumor growth by supplying nutrients and oxygen and eliminating metabolic waste [1]. Therefore, anti-angiogenic therapeutics targeting tumor endothelial cells (ECs) have been widely explored for anti-cancer therapy [2]. To date, many endogenous anti-angiogenic proteins or protein fragments have been discovered, and the list is still growing [3–5].

A Disintegrin and Metalloproteinase with Thrombospondin Motifs 5 (ADAMTS5), also known as aggrecanase-2, is a 100 kDa matrix metalloproteinase of the ADAMTS family. It is the major aggrecanase involved in aggrecan degradation of the articular cartilage in human osteoarthritis [6, 7]. ADAMTS5 is synthesized as a zymogen and secreted through the endoplasmic reticulum secretory pathway via its signal peptide. Secreted ADAMTS5 zymogen gets cleaved by furin through extracellular processing to become an active extracellular matrix (ECM) proteinase. Further C-terminal autocatalytic cleavages generate two truncated fragments with molecular weights of 45 kDa (TS5-p45) and 60 kDa (TS5-p60), respectively, with both truncate retaining the metalloproteinase activity of ADAMTS5 [8, 9]. In addition to aggrecan, ADAMTS5 also cleaves other proteoglycans such as brevican (contributing to glioblastoma in brain tissue [10]) and versican (contributing to various vascular diseases [11] and embryonic development [12–14]).

We previously reported a novel role of ADAMTS5 as an angiogenesis inhibitor, suppressing tumor growth [15, 16]. In the subcutaneous xenograft mouse tumorigenesis model, B16 melanoma cells overexpressing full-length ADAMTS5 and TS5-p45 truncate both suppressed tumor angiogenesis and enhanced apoptosis within the tumor, resulting in tumor growth suppression. We further demonstrated that the anti-angiogenic/anti-cancer activity of ADAMTS5 is independent of its metalloproteinase activity but is mediated through its central thrombospondin type 1 repeat domain (TSR1) [15–17]. Furthermore, this anti-cancer activity correlated with the downregulation of pro-angiogenic factors such as vascular endothelial growth factor (VEGF) and platelet-derived endothelial cell growth factor (PD-ECGF) in tumor tissues. Since TS5-p45 recapitulates the anti-angiogenic activity of ADAMTS5, we used recombinant TS5-p45 to demonstrate that TS5-p45 dose-dependently triggers apoptosis of human umbilical vein endothelial cells (HUVECs) in a caspase-dependent manner [15]. This pro-apoptotic activity most likely is responsible for the anti-angiogenic function of ADAMTS5. However, how ADAMTS5 triggers EC apoptosis and what the EC receptor targeted is unclear.

Nucleolin (NCL) is a ubiquitously expressed multifunctional protein predominantly localized in the nucleolus and accounts for up to 10% of the total protein in the nucleolar region. It is also present in the nucleoplasm, the cytoplasm, and the cell membrane [18–20]. While its nucleolar expression correlates with the level of ribosomal activity and cell proliferation, NCL is translocated to the EC surface during angiogenesis and is a marker for angiogenic blood vessels [20–22]. This EC surface translocation of NCL is stimulated by VEGF and mediated by non-muscle myosin. Cell surface NCL is also upregulated in many types of cancer cells and is localized at the external side of the plasma membrane [23, 24]. Notably, cell surface NCL forms receptor-ligand complexes with several extracellular anti-tumorigenic/anti-angiogenic proteins, including endostatin [25], kallistatin [26], the tumor-homing F3 peptide [24], and pseudopeptides HB-19 [27] and N6L [28]. Furthermore, cell surface NCL shuttles its extracellular ligands to the cytoplasm and nucleus via internalization in multiple types of cancer cells and tumor ECs [24, 29–31].

This work identifies cell surface NCL as a novel receptor for ADAMTS5 that mediates its pro-apoptotic activity in ECs. Furthermore, we show that NCL rapidly shuttle extracellular TS5-p45 to the nucleus and this nuclear translocation is critical for TS5-p45 to induce EC apoptosis.

## Materials and methods

### Cell culture and reagents

All cells were cultured at 37 °C incubators with 5% CO_2_. HUVECs were purchased from Merck (SCCE001) and cultured on 0.2% gelatin-coated culture flasks in EndoGRO-LS complete culture media (Merck, SCME001). Early HUVEC passage numbers (passages 4–8) were used. Human embryonic kidney 293T (HEK293T) cells were cultured in Dulbecco’s modified Eagle’s medium (DMEM) supplemented with 10% fetal bovine serum (FBS; Cytiva, SV30160.03). Recombinant human TS5-p45 (C-terminal His-tagged, Ser262-Pro622 truncate of ADAMTS5) was purchased from R&D Systems (2198-AD-01M). Recombinant human NCL (FLAG-tagged, full length) was purchased from OriGene (TP319082).

### Apoptosis measurement using Incucyte

Apoptosis was determined using Incucyte Zoom Live-Cell Analysis System (Essen Bioscience, Sartorius). Briefly, HUVECs at a cell density of 1 × 10^4^ cells/well were seeded in a 96-well plate for overnight incubation and starved in EndoGRO Basal Media (BM; contains 2% FBS) for 3 h (h) on the following day. Apoptosis was measured using CellEvent^TM^ Caspase-3/7 Green Detection Reagent (Invitrogen, C10423). Cells were treated with TS5-p45 for 24 h in the presence of 20 ng/ml VEGF_165_ (R&D Systems, 293-VE-010). Apoptosis inhibitors (caspase-8 (Cas-8) inhibitor, Z-IETD-FMK (R&D Systems, FMK007) and caspase-9 (Cas-9) inhibitor, Z-LEHD-FMK (R&D Systems, FMK008)) were pre-incubated with HUVECs for 30 min (min) before proceeding to TS5-p45 treatment. The endosome-destabilizing peptide L17E (GenScript, IWLTALKFLGKHAAKHEAKQQLSKL-CONH_2_) was applied to HUVECs for 1 h before proceeding to TS5-p45 treatment [32]. Untransfected control (Ctrl), control siRNA (siCtrl)- and NCL-specific siRNA (siNCL)-transfected HUVECs were seeded after 48 h post-siRNA transfection for apoptosis induction by TS5-p45.

### Caspase activity measurement

HUVECs treated with 250 nM TS5-p45 for 24 h, 10 μg/ml Etoposide (ETOP; Sigma Aldrich, E1383) for 24 h and 0.5 μM Staurosporine (STS; Sigma Aldrich, S6942) for 6 h, respectively, were harvested for isolation of total protein lysate. Protein concentrations were quantified by Bradford assay. Colorimetric Cas-8 and Cas-9 activity measurements were performed with Caspase-8 Assay Kit (Abcam, ab39700) and Caspase-9 Assay Kit (Abcam, ab65608) according to the manufacturer’s protocol. Protein lysate at 50 μg/well was incubated with Cas-8 substrate (IETD-pNA) and Cas-9 substrate (LEHD-pNA), respectively, at 37 °C overnight. The plate was read at 405 nm with Hidex Sense Microplate Reader (Hidex, Finland), and buffer-related background readings were subtracted from sample readings.

### Mitochondrial membrane potential (ψ_mito_) measurements

For detection of ψ_mito_, HUVECs were treated with 250 nM TS5-p45 for 24 h, followed by incubation with serum-free media containing 200 nM MitoTracker^TM^ Green FM (Invitrogen^TM^, M7514) and 600 nM Tetramethylrhodamine Methyl Ester Perchlorate (TMRM; Invitrogen^TM^, T668) for 30 min before imaging with FV3000 Confocal Laser Scanning Microscope (Olympus, Japan). Untreated HUVECs were used as control. Dynamic measurement of ψ_mito_ was performed with HUVECs at a cell density of 1 × 10^4^ cells/well in a 96-well plate pre-treated with 600 nM TMRM at 37 °C for 30 min and subsequently treated with 250 nM TS5-p45 treatment at 37 °C for 24 h in the presence of 600 nM TMRM. TMRM fluorescence signal was measured using Incucyte Zoom (Essen Bioscience, Sartorius), and TMRM signal at 24 h post-treatment was normalized to TMRM signal at 0 h. Carbonyl cyanide 3-chlorophenylhydrazone (CCCP; Sigma Aldrich, C2759) at 10 μM was used as a positive control to disrupt ψ_mito._

### Human apoptosis antibody array

HUVECs treated with 250 nM TS5-p45 for 24 h were harvested for isolation of total protein lysate. Protein concentrations were quantified by Bradford assay, and 250 µg total protein lysate/array was used. Proteome Profiler Human Apoptosis Array Kit (R&D Systems, ARY009) was used according to the manufacturer’s protocol. Arrays were scanned with Amersham Imager 600 (GE Healthcare, USA), and analysis for band intensity comparison was done by ImageJ.

### Western blot

HUVECs after transfections/treatments were lysed by either RIPA buffer for whole cell lysate (WCL) or cell fractionation buffers (Abcam, ab109719) for subcellular fractions. Protein samples were then electroporated, transferred, and incubated with respective near-infrared fluorescent dye-conjugated secondary antibodies (LI-COR Biosciences, USA), followed by scanning with Odyssey CLx Imaging System (LI-COR Biosciences, USA). Primary antibodies for Western Blot (WB) were purchased from Santa Cruz Biotechnology, including anti-Cytochrome C (A-8, sc-13156), anti-GAPDH (6C5, sc-32233), anti-Lamin A/C (636, sc-7292), anti-HSP60 (H-1, sc-13115), anti-TOM20 (F10, sc-17764), anti-VE-cadherin (C19, sc-6458) and Sigma-Aldrich including anti-FLAG (M2, F1804). Antibodies against His-tag and NCL were from GenScript (anti-His, A00186) and Abcam (anti-His, ab9108, and anti-NCL, ab22758). Secondary antibodies for WB were purchased from LI-COR Biosciences, including donkey anti-goat IgG IRDye 800CW (926-32214), donkey anti-mouse IgG IRDye 680RD (926-68072), donkey anti-mouse IgG IRDye 800CW (926-32212), and donkey anti-rabbit IgG IRDye 800CW (926-32213).

### Internalization assays

HUVECs were treated with various concentrations of TS5-p45 for 1 h and with 250 nM TS5-p45 for 5, 10, 15, 40, 45, and 60 min, respectively. Following the treatments, cells were washed with acidic PBS (pH 3.5) twice and normal PBS (pH 7.2) twice to remove cell surface-bound TS5-p45 and subjected to WB analysis. For endocytosis study, cells were pre-treated with various endocytosis pathway inhibitors, including Dynasore (DYN), Wortmannin (WORT), Chlorpromazine (CPZ), and Nystatin (NYS) for 1 h, followed by 250 nM TS5-p45 treatment for another 1 h. For L17E experiments, HUVECs were pre-treated with L17E for 1 h, followed by 250 nM TS5-p45 treatment for another 1 h. For TS5-p45 internalization under NCL knockdown, Ctrl, siCtrl- and siNCL-transfected HUVECs (48 h post-transfection) were treated with 250 nM TS5-p45 for 1 h, respectively. For Ivermectin experiments, HUVECs were pre-treated with Ivermectin for 1 h, followed by 250 nM TS5-p45 treatment for another 1 h.

### Subcellular fractionations

The plasma membrane fractions (MF) of Ctrl, siCtrl- and siNCL-transfected HUVECs (48 h post-transfection) were isolated as previously described [25]. HUVECs were treated with 250 nM TS5-p45 for 1 h and 24 h, respectively, or with various pre-treatments/transfections including L17E peptide for 1 h, siCtrl- and siNCL-transfections for 48 h, cell surface biotinylation for 30 min at RT and Ivermectin for 1 h, followed by 250 nM TS5-p45 treatment for 1 h, respectively. Following the treatments, subcellular fractions (cytoplasmic (Cyto), mitochondrial (Mito), and nuclear (Nuc) fractions) were obtained using Cell Fractionation Kit (Abcam, ab109719) according to the manufacturer’s protocol.

### Identification of EC surface receptor for TS5-p45

EC surface receptor for ADAMTS5 was identified by pulldown experiments using TS5-p45 as the bait and EC plasma membrane extract as the receptor source in a similar fashion as previously described [33]. Briefly, 10 μg of recombinant His-tagged TS5-p45 was incubated with 300 μg of EC plasma membrane extract and 50 μl of anti-His microbeads at 4 °C overnight. Magnetic isolation was performed using µMACS His Isolation Kit (Miltenyi Biotec, 130-091-124) according to the manufacturer’s protocol. TS5-p45 interacting complex was then separated by SDS-PAGE, and interacting membrane proteins appearing as unique bands under TS5-p45-incubated lane were identified by MALDI-TOF-TOF MS (AB SCIEX, Framingham, MA, USA). Receptor-ligand binding was then verified by two-way co-IP using purified recombinant candidate proteins such as NCL and TS5-p45. Receptor function is verified by siRNA knockdown followed by apoptosis assay.

### siRNA transfection

HUVECs were grown to 60–70% in confluence, and 5 × 10^5^ cells/reaction were harvested and prepared for nucleofection. Human Umbilical Vein Endothelial Cell Nucleofector^TM^ Kit (Lonza, VPB-1002) was used according to the manufacturer’s protocol with Nucleofector^TM^ 2b Device (Lonza, AAB-1001). siRNAs used are: NCL sense: 5ʹ-GGAUAGUUACUGACCGGGAAACUGG-3ʹ and NCL anti-sense: 5ʹ-CCAGUUUCCCGGUCAGUAACUAUCCUU-3ʹ. Control siRNA (Negative Control DsiRNA, 51-01-14-03) was purchased from Integrated DNA Technologies (IDT). Cells were harvested 48 h post-transfection for all analyses.

NCL mammalian expression constructs cloned into pCMV-Tag2 vector (Agilent, 211172) with N-terminal FLAG tag obtained from Professor Yuki Kuwano [34]. Overexpression of NCL and truncates in HEK293T cells were achieved via transient transfection using branched polyethylenimine (bPEI) (Sigma Aldrich, 408727). HEK293T cells were transfected with expression plasmid DNA, and cells were harvested at 48 h post-transfection. WCL was generated using lysis buffer (150 mM NaCl, 1% Triton X-100, 50 mM Tris-HCl pH 7.4) and analyzed by WB.

### Immunofluorescence staining

For detection of TS5-p45 intracellular trafficking, HUVECs seeded on coverslips were treated with 250 nM TS5-p45 at 37 °C for 1 h, fixed with 4% paraformaldehyde (PFA) (Sigma-Aldrich) in PBS at room temperature for 20 min, and permeabilized with 0.3% Triton X-100/PBS at room temperature for 15 min. After blocking with 3% BSA/PBS at room temperature for 1 h, cells were incubated with mouse anti-His (GenScript, A00186) at 4 °C overnight. The following day, cells were incubated with goat anti-mouse IgG, Alexa Fluor 488 (Invitrogen, A-11029) at room temperature for 2 h before being mounted with VECTASHIELD^TM^ Mounting Medium with 4,6-diamidino-2-phenylindole (DAPI) (Vector Laboratories, USA). For detection of surface-bound TS5-p45 and its dual staining with NCL, cells were fixed in 2% PFA solution at room temperature and then subjected to anti-NCL binding (Abcam, ab22758) at room temperature for 2 h before they were washed with PBST thrice. Cells were then incubated with 250 nM TS5-p45 at room temperature for 2 h, followed by washing, blocking, and incubating with mouse anti-His at 4 °C overnight. The following day, cells were washed and incubated with goat anti-mouse IgG, Alexa Fluor 488 (Invitrogen, A-11029), and goat anti-rabbit IgG, Alexa Fluor 568 (Invitrogen, A-11011), respectively. Fluorescent images were acquired with FV3000 Confocal Laser Scanning Microscope (Olympus, Japan) and processed with ImageJ.

### Cellular ELISA

HUVECs at a cell density of 1 × 10^4^ cells/well in a 96-well plate were fixed with 4% PFA at room temperature for 10 min. Non-permeabilized cells were blocked with 3% BSA/PBS at room temperature for 2 h before they were treated with various concentrations of TS5-p45 at room temperature for 2 h. Following the treatment, cells were washed with PBST thrice at room temperature for 5 min each and incubated with anti-His (Abcam, ab9108) and isotype control anti-IgG antibody (Santa Cruz, sc2027) incubations at 4 °C overnight. The next day, cells were washed as described and incubated with goat anti-rabbit IgG, Alexa Fluor 488 (Invitrogen, A-11034) at room temperature for 2 h, before fluorescence intensity was measured with Hidex Sense Microplate Reader (Hidex, Finland). Next, cells were counter-stained with 1 µg/ml DAPI (Calbiochem, Sigma-Aldrich, 268298) in PBS at room temperature for 10 min, followed by washing as described. Fluorescence intensity measurements for DAPI were used to normalize TS5-p45 fluorescence intensity across all conditions.

### Flow cytometry

HUVECs were harvested in 1 mM EDTA/PBS and allocated as 2.5 × 10^5^ cells/sample. Unfixed non-permeabilized cells were blocked with 3% BSA/PBS on ice for 30 min, followed by incubation with TS5-p45 at 4 °C for 4 h. Following the treatment, cells were washed with 1% BSA/PBS thrice and incubated with FITC conjugated anti-His (Miltenyi Biotec, 130-123-633) and anti-IgG (Miltenyi Biotec, 130-113-761) (both in 1%BSA/PBS) along with LIVE/DEAD^TM^ Fixable Red Dead Cell Stain (Invitrogen, L23102), respectively at 4 °C for 30 min. After washing, 10,000 cells/sample were immediately analyzed with CytoFLEX LX Flow Cytometer (Beckman Coulter, USA), and results were prepared in the instrument’s built-in software. The same protocol was performed with the same number of cells for Ctrl, siCtrl- and siNCL-transfected HUVECs, respectively.

### Surface plasmon resonance (SPR)

SPR was performed using a Biacore T200 instrument (Cytiva, USA) with a Series S CM5 sensor chip (Cytiva, BR100399), at 25 °C, with HEPES-buffered saline with Tween-20 (HBST: 10 mM HEPES, 150 mM NaCl, 50 µM EDTA, pH 7.4, 0.05% Tween-20) as running buffer. Anti-NCL antibody (Abcam, ab22758) was first immobilized using the amine-coupling method. The surfaces of flow cells (Fc) 1 and 2 were activated for 7 min with a 1:1 mixture of 0.1 M NHS and 0.1 M EDC. 50 µg/mL anti-NCL antibody in 10 mM sodium acetate pH 5.0 was then injected over Fc1 and Fc2 for 7 min. The surfaces were then deactivated by injecting 1 M ethanolamine pH 8.0 for 7 min. The final immobilization levels of the antibody were 10,000–11,000 response units (RU). After overnight equilibration of the sensor surfaces, a single-cycle kinetics method was employed to investigate NCL binding to TS5-p45. Recombinant NCL protein was captured on Fc2 but not Fc1 by injecting 10 µg/ml NCL in running buffer at a flow rate of 10 µl/min for 120 s. Fc1 was left blank to serve as a reference surface. Five serial dilutions of TS5-p45 in HBST buffer were injected over the two flow cells at 30 μl/min for 120 s (s). Dissociation was measured for 600 s before the surface was regenerated by using 10 mM glycine pH 1.5. A buffer-only reference cycle was performed by using 5 injections of running buffer under identical conditions. All sensorgram data were collected at a rate of 10 Hz. Double-referenced sensorgrams were analyzed and fitted to the 1:1 binding model using the Biacore T200 Evaluation Software version 3.0.

### ELISA saturation binding

Anti-FLAG antibody-coated plate (GenScript, L00455C), recombinant His-tagged TS5-p45, and FLAG-tagged NCL-FL and Ctrl overexpression WCL were used in this experiment. Specific FLAG-tagged NCL-FL capture was first verified by titrating the Anti-FLAG antibody-coated plate with increasing doses of NCL-FL lysate. Anti-NCL primary antibody (Abcam, ab22758) and Alexa Fluor 488 conjugated anti-rabbit IgG (Invitrogen, A-11034) were used for detection. The same protocol was applied to Ctrl lysates as a negative control. Saturation binding experiment was then performed by titrating TS5-p45 over FLAG-tagged NCL-FL- and Ctrl-captured wells at 4 °C for overnight incubation. The following day, treated wells were incubated with anti-His (Abcam, ab9108) at room temperature for 2 h and Alexa Fluor 488 conjugated anti-rabbit IgG (Invitrogen, A-11034) at room temperature for 1 h. Finally, fluorescence intensity was measured with Hidex Sense Microplate Reader (Hidex, Finland). Binding affinity was calculated with GraphPad Prism 8.

### Immunoprecipitation

NCL full-length and truncates were transiently transfected into HEK293T cells, and WCL was generated. FLAG-tagged NCL truncates (2 mg WCL/reaction) and His-tagged TS5-p45 (1 µg/reaction), respectively, were incubated at 4 °C overnight and subjected to IP experiments using EZview^TM^ Red ANTI-FLAG^®^ M2 Affinity Gel (Sigma-Aldrich, F2426) according to the manufacturer’s protocol. WB antibodies used in these experiments were anti-FLAG (Sigma-Aldrich, F1804) and anti-His (Abcam, ab9108).

### Cell surface biotinylation and streptavidin pulldown

EZ-Link^TM^ Sulfo-NHS-LC-Biotin (Thermo Scientific, 21335) was used to biotinylate the cell surface proteins of intact HUVECs according to the manufacturer’s protocol. Biotinylated cells were then treated with 250 nM TS5-p45 for 1 h and lysed as WCL or fractionated as described. To eliminate sticky DNA content in Nuc fractions, the fractions were subjected to 250 U/ml Benzonase Nuclease treatment (Millipore, E1014) at 4 °C for 1 h. Fractions were then centrifuged at 10,000 × *g* at 4 °C for 10 min to obtain DNA/RNA-free Nuc fractions. Pierce™ Streptavidin Magnetic Beads (Thermo Scientific, 88817) were used to precipitate the biotinylated proteins along with their binding partners from WCL and Nuc fractions, respectively, according to the manufacturer’s protocol with DynaMag^TM^-2 Magnet (Invitrogen, 12321D). Eluted samples were then analyzed by WB. For CaCl_2_ and EGTA pre-treatments experiments, cells were pre-treated with 3 mM CaCl_2_ and 5 mM EGTA for 1 h, respectively, followed by biotinylation, TS5-p45 treatment, WCL harvesting, Streptavidin pulldown, and WB analysis as described.

### RNA-seq and qRT-PCR

HUVECs treated with 250 nM TS5-p45 for 12 h were harvested with cold TRIzol^TM^ reagent (Invitrogen^TM^, 15596018) for total RNA isolation according to the manufacturer’s protocol. Total RNA of 1 μg sample/condition was used for RNA-seq performed by Novogene Singapore. Protein-coding genes with a mean count per million (cpm) bigger than 2 were included in differentially expressed gene (DEG) analysis using DESeq2 [35]. Genes with at least two-fold change at both directions (TS5-p45 vs. Ctrl) with adjusted *p*-values less than 0.05 were selected as valid DEGs. DEGs that overlapped with the hallmark apoptosis gene set from MsigDB (http://www.gseamsigdb.org/gsea/msigdb/index.jsp) were analyzed. Two-step qRT-PCR was performed for Ctrl, TS5-p45, and siNCL+TS5-p45 samples using CFX96 Touch Real-Time PCR System and SsoAdvanced™ Universal SYBR^®^ Green Supermix (2x) (Bio-Rad, USA) with the primer sets listed in Table S1. *GAPDH* was used as a housekeeping gene for normalization, and the gene expression levels were calculated using the 2^−ΔΔCt^ formula with respect to the Ctrl samples.

### Statistical analysis

Statistical differences were evaluated using one-way or two-way analysis of variance (ANOVA) for more than two groups and a two-tailed paired t-test for two groups as described in each figure legend. *p* < 0.05 was considered statistically significant. Results are presented as mean ± standard deviation (SD).

### Data deposition

The RNA-seq data have been deposited into NCBI GEO with accession number: GSE191049.

## Results

### TS5-p45 induces EC apoptosis through both Cas-8 and Cas-9 without affecting mitochondrial integrity

**Fig. 1:**
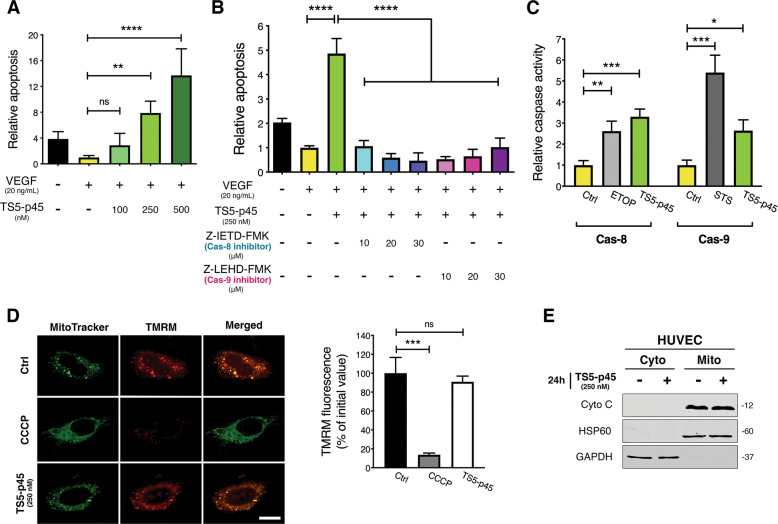
TS5-p45 induces EC apoptosis through both Cas-8 and Cas-9 without affecting mitochondrial integrity. **A** TS5-p45 induces EC apoptosis in a dose-dependent manner. Apoptotic activity in ECs was measured at 24 h post-TS5-p45 treatment. Data shown are mean ± SD from three independent experiments in triplicates. **B** Cas-8 and Cas-9 inhibitors both impaired the TS5-p45-induced apoptosis. HUVECs pre-treated with Cas-8 inhibitor (Z-IETD-FMK) and Cas-9 inhibitor (Z-LEHD-FMK) were treated with TS5-p45, and apoptosis was measured at 24 h post-TS5-p45 treatment. Data shown are mean ± SD from three independent experiments in triplicates. **C** TS5-p45 induces both Cas-8 and Cas-9 activities. HUVEC lysates obtained at 24 h post-TS5-p45 treatment were used to determine respective caspase activities. ETOP- and STS-treated lysates were used as positive controls for Cas-8 and Cas-9 activities, respectively. Data shown are mean ± SD from three independent experiments in duplicates. **D** ψ_mito_ is not disturbed by TS5-p45. ψ_mito_ is indicated by TMRM staining, and MitoTracker green is used to label mitochondria. Scale bar represents 10 μm. TMRM signal from HUVECs at 24 h post-TS5-p45 treatment was compared to TMRM signal at 0 h. CCCP treatment was used as a control to disrupt ψ_mito_. Data shown are mean ± SD from three independent experiments in triplicates. **E** TS5-p45 does not induce Cyto C release into the cytosol. Cyto: cytosol, Mito: mitochondria. Molecular weight (kDa) is indicated on the right side of the WB. Statistical analysis was performed by one-way ANOVA. ns, not significant; *p < 0.05; **p < 0.01; ***p < 0.001; ****p < 0.0001.

TS5-p45, containing residues 262-622 of human ADAMTS5, functions similarly as the full-length ADAMTS5 as an anti-angiogenic protein both in vitro and in vivo. In addition, TS5-p45 induced EC apoptosis in a caspase-dependent manner [15]. In this work, we further investigated the apoptosis pathways involved in TS5-p45-induced EC apoptosis. TS5-p45 induced potent EC apoptosis under both basal endothelial cell culture media and basal media + VEGF (Fig. S1A). Similar to our previous reports, all subsequent apoptosis studies were done under basal media+VEGF [15]. Based on the dose-dependent EC apoptosis induced by TS5-p45 (Fig. 1A), we chose 250 nM TS5-p45 as a potent pro-apoptotic concentration for this study. Dynamic monitoring showed that TS5-p45-induced apoptosis could first be detected around 8 h post-treatment and steadily increased in a time-dependent manner (Fig. S1B).

The extrinsic apoptosis pathway facilitated by Cas-8 and the intrinsic pathway (through mitochondria) facilitated by Cas-9 lead to activation of downstream effector Cas-3, leading to apoptosis [36]. Furthermore, we found that specific inhibitors for both Cas-8 and Cas-9 potently diminished TS5-p45-induced EC apoptosis (Fig. 1B), while the inhibitors alone did not harbor any pro-apoptotic activity (Fig. S2A). Indeed, TS5-p45 treatment triggered the enzymatic activations of both Cas-8 and Cas-9 (Fig. 1C and Fig. S2B). However, the mitochondrial membrane potential ψ_mito_ measured by TMRM fluorescent staining was not perturbed at 24 h post-TS5-p45 treatment when apoptosis is already full-blown, indicating that mitochondria membrane integrity is intact (Fig. 1D). Consistently, no cytochrome C (Cyto C) was released from mitochondria to cytosol under TS5-p45 (Fig. 1E).

Further examination of proteins in the apoptosis signaling pathways using a human apoptosis antibody array showed that TS5-p45 treatment led to a 6.9-fold upregulation of cleaved Cas-3 (cCas-3) (Fig. S2C, D), validating the activation of the caspase-dependent apoptosis pathway. Meanwhile, no alteration in Cyto C level was observed (Fig. S2C). Other proteins that showed ≥ 1.5 fold upregulation include TRAILR2 (from the extrinsic apoptosis pathway) and HTRA2, SMAC, and XIAP (from the intrinsic apoptosis pathway). In addition, upregulation of heat shock proteins, HSP32 and HSP60, which are modulators of apoptosis/survival, as well as HIF-1α, a transcription factor involved in hypoxia-mediated apoptosis, are observed. However, how these proteins are involved in TS5-p45 mediated EC apoptosis is not clear at this moment.

### NCL is a high-affinity cell surface receptor for TS5-p45 in ECs

**Fig. 2:**
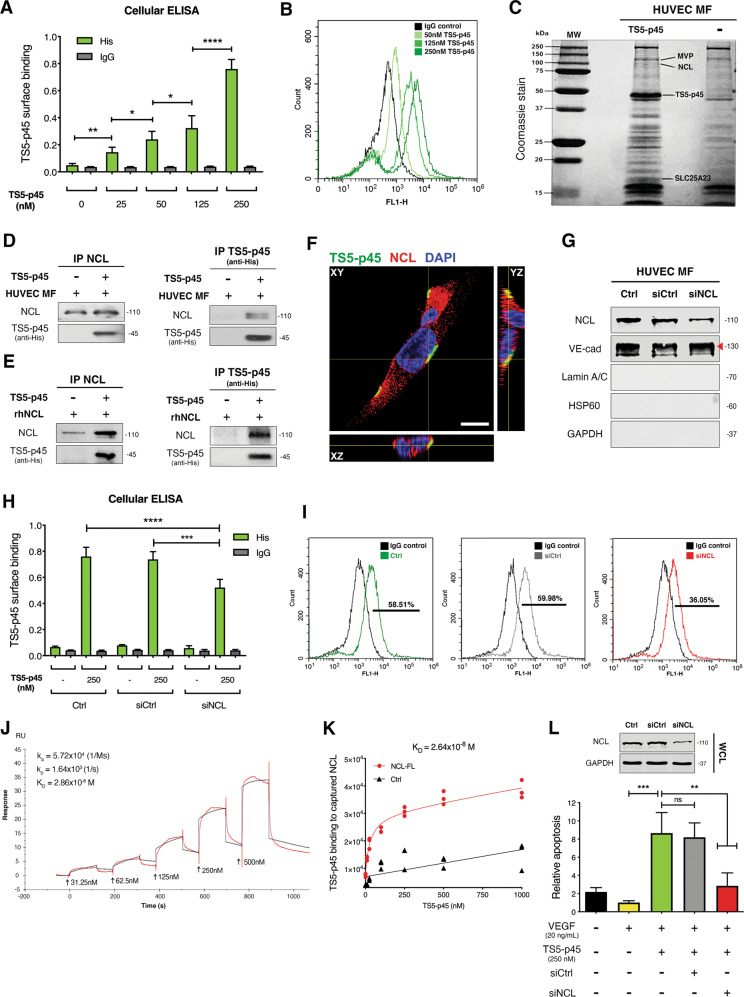
NCL is a high-affinity cell surface receptor for TS5-p45 in ECs. **A**, **B** TS5-p45 binds to the HUVEC surface in a dose-dependent manner. Intact HUVECs were treated with various doses of TS5-p45, and surface-bound TS5-p45 was measured through **A** cellular ELISA by anti-His detection and **B** flow cytometry by anti-His-FITC detection. IgG, isotype control antibody. Cellular ELISA data shown are mean ± SD from two independent experiments in triplicates. In flow cytometry experiments, anti-IgG-FITC signal was used to set the threshold. The representative set is shown in overlay histogram format. Flow cytometry gating applied for this experiment is shown in Fig. S4. **C**–**E** NCL directly binds TS5-p45. **C** TS5-p45 binding partners on HUVEC MF were identified by pulldown for subsequent MS analysis. **D** Two-way co-IP using TS5-p45 (anti-His) and HUVEC MF showed TS5-p45 and NCL binding. **E** Two-way co-IP using recombinant TS5-p45 and recombinant NCL demonstrating direct binding between TS5-p45 and NCL. **F** Confocal microscopy showing co-localization of surface-bound TS5-p45 with endogenous cell surface NCL. Anti-His (TS5-p45, green), anti-NCL (red), and DAPI (Nuc marker, blue) triple staining and the three-planar view of cells were shown. Scale bar represents 5 μm. **G** siRNA knockdown reduced cell surface expression level of NCL in HUVECs. siNCL-transfected HUVECs were analyzed by anti-NCL WB, with VE-cadherin used as a loading control for MF. WB showed the purity of the MF with anti-Lamin A/C (Nuc marker), anti-HSP60 (Mito marker), and anti-GAPDH (Cyto marker). **H**, **I** Cell surface-bound TS5-p45 is reduced upon siNCL knockdown. siRNA-transfected HUVECs were treated with TS5-p45, and cell surface-bound TS5-p45 was measured through **H** cellular ELISA by anti-His detection and **I** flow cytometry by anti-His-FITC detection. IgG, isotype control antibody. Cellular ELISA data shown are mean ± SD from two independent experiments in triplicates. In flow cytometry experiments, anti-IgG-FITC signal was used to set the thresholds, respectively. Data from a representative set is shown in separate overlay histograms. Gating applied for this flow cytometry experiment is shown in Fig. S5. **J**, **K** Determination of binding affinity between NCL and TS5-p45. **J** SPR sensorgrams from TS5-p45 as analyte over NCL-immobilized chip surface. Red and black curves represent the experimental data and fit to the 1:1 model of the interaction, respectively, with the binding constants indicated. **K** Binding affinity determined by ELISA. Saturation binding of TS5-p45 to NCL-captured wells was performed. Each treatment concentration/data point is the average of triplicate readings. **L** NCL is a functional receptor for TS5-p45-induced apoptosis. siRNA-transfected HUVECs with reduced NCL expression were treated with TS5-p45 and apoptosis measured at 24 h post-treatment. Relative apoptosis is normalized to the VEGF-containing condition. Data shown are mean ± SD from three independent experiments in triplicates. Statistical analysis was performed by one-way ANOVA. ns not significant; *p < 0.05; **p < 0.01; ***p < 0.001 and ****p < 0.0001.

To determine if the extracellular TS5-p45 triggers EC apoptosis by interacting with an EC cell surface receptor, intact HUVECs were treated with various concentrations of TS5-p45 (His-tagged) on ice and stained with anti-His antibody to detect the surface-bound TS5-p45. As shown in Fig. 2A, B and Fig. S3, cellular ELISA and flow cytometry analyses showed that TS5-p45 dose-dependently binds to EC surface while control IgG showed no such binding. These results suggest that there is a cell surface receptor for ADAMTS5 in ECs.

Previously, Yamamoto K et al. reported that low-density lipoprotein receptor-related protein 1 (LRP1) is a cell surface receptor for ADAMTS5 in chondrocytes [37]. However, the LRP1 protein level was negligible in HUVECs (data not shown), suggesting that ECs harbor a different receptor for ADAMTS5. To identify the EC surface receptor for ADAMTS5, TS5-p45 was used as a bait to interact with EC membrane fraction (MF), and proteins that interact with TS5-p45 were pulled down by magnetic anti-His microbeads. TS5-p45, together with bound proteins, were subjected to SDS-PAGE, and unique protein bands in the TS5-p45-treated sample were analyzed by Mass Spectrometry (MS). Three potential TS5-p45-interacting proteins with significant scores were identified: major vault protein (MVP), NCL, and solute carrier 25A23 (SLC25A23) (Fig. 2C). Co-IP experiments using TS5-p45 and HUVEC MF confirmed that TS5-p45 interacted with MVP and NCL, but not with SLC25A23 (Fig. 2D and Fig. S4A, B), suggesting that MVP and NCL are receptor candidates. Subsequently, co-IP experiments in both directions using recombinant human NCL (rhNCL) and MVP (rhMVP) proteins revealed that TS5-p45 directly interacts with NCL (Fig. 2E), but not with MVP (Fig. S4C). These results suggest that NCL is the EC surface receptor for TS5-p45.

While NCL is one of the major proteins of the nucleolus, it is also expressed on the cell surface, where it serves as a receptor for multiple ligands implicated in angiogenesis and tumorigenesis [24]. To verify that cell surface NCL is indeed the functional receptor mediating TS5-p45’s binding to HUVEC cell surface and its pro-apoptotic function, we used confocal fluorescence microscopy to demonstrate that TS5-p45 co-localizes with NCL on EC surface after incubating with HUVECs (Fig. 2F). NCL knockdown using siRNA transient transfection reduces cell surface NCL level by ~40–50% (Fig. 2G). Meanwhile, TS5-p45’s binding to the EC surface was suppressed by ~40% in siNCL-transfected HUVECs, correlating with the reduction of cell surface NCL (Fig. 2H, I; Figs. S5, S6). These results support that NCL is the receptor mediating the binding of TS5-p45 to the EC surface.

Receptor-ligand interactions play a critical role in cell signal transduction, with their binding affinities determining the specificity and selectivity of receptor activation. Therefore, we used SPR and ELISA saturation binding approaches to study the binding kinetics and affinity between TS5-p45 and NCL. Using SPR, in which anti-NCL antibody was first coupled to the chip surface, and recombinant NCL was then immobilized onto the chip surface via antibody binding, the binding affinity between NCL and TS5-p45 is determined to be around 28 nM (Fig. 2J). Moreover, an ELISA binding method was also used to determine the binding affinity between NCL and TS5-p45. In this case, FLAG-tagged full-length NCL (NCL-FL) expressed in transiently transfected HEK293T cells was first attached to anti-FLAG-coated 96-well plates, and dose-dependent NCL capture with saturation profile was observed (Fig. S7). Saturation binding of TS5-p45 to the anchored NCL showed a binding affinity around 26 nM (Fig. 2K), consistent with the affinity calculated based on the SPR approach. These results indicate that TS5-p45 binds to NCL with relatively high affinity.

More importantly, when total NCL was knocked down by about 70% in HUVECs by siNCL transient transfection, a significant reduction in TS5-p45-induced apoptotic activity was observed (Fig. 2L). Altogether, these observations demonstrated that cell surface NCL is a novel functional receptor for ADAMTS5 and mediates TS5-p45-induced EC apoptosis.

### NCL binds to TS5-p45 through its RNA binding domains (RBDs)

**Fig. 3:**
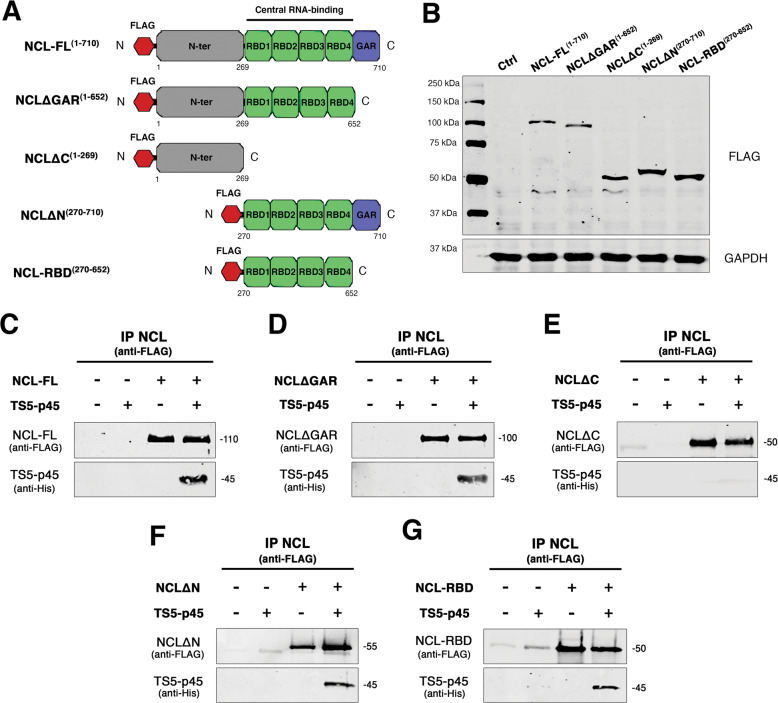
NCL binds to TS5-p45 through its RNA binding domains (RBDs). **A** Schematic representation of NCL expression constructs with N-terminal FLAG tag encoding NCL-FL^1-710^, NCLΔGAR^1-652^, NCLΔC^1-269^, NCLΔN^270-710^, and NCL-RBD^270-652^. **B** Expression of NCL and truncates in HEK293T cells by WB via anti-FLAG antibody. GAPDH was used as a loading control. **C**–**G** Co-IP analyses of FLAG-tagged NCL and truncates against His-tagged TS5-p45. Binding between TS5-p45 and **C** NCL-FL^1-710^, **D** NCLΔGAR^1-652^, **E** NCLΔC^1-269^, **F** NCLΔN^270-710^, **G** NCL-RBD^270-652^ were shown by anti-FLAG and anti-His blotting, respectively.

NCL is a multidomain protein containing an N-terminal domain, four central RBDs, and a C-terminal GAR domain [38]. To determine which NCL domain mediates its binding to TS5-p45, we obtained FLAG-tagged NCL, and NCL truncates from transiently transfected HEK293T cells (Fig. 3A, B) and performed co-IP experiments using anti-FLAG affinity beads and TS5-p45. As shown in Fig. 3C–G, NCL-FL, NCLΔGAR, NCLΔN, and NCL-RBD bind to TS5-p45, with only NCLΔC losing the ability to bind TS5-p45. These results revealed that the RBD domain is essential and sufficient for NCL to bind TS5-p45.

### TS5-p45 is internalized and trafficked to the nucleus upon interacting with cell surface NCL

**Fig. 4:**
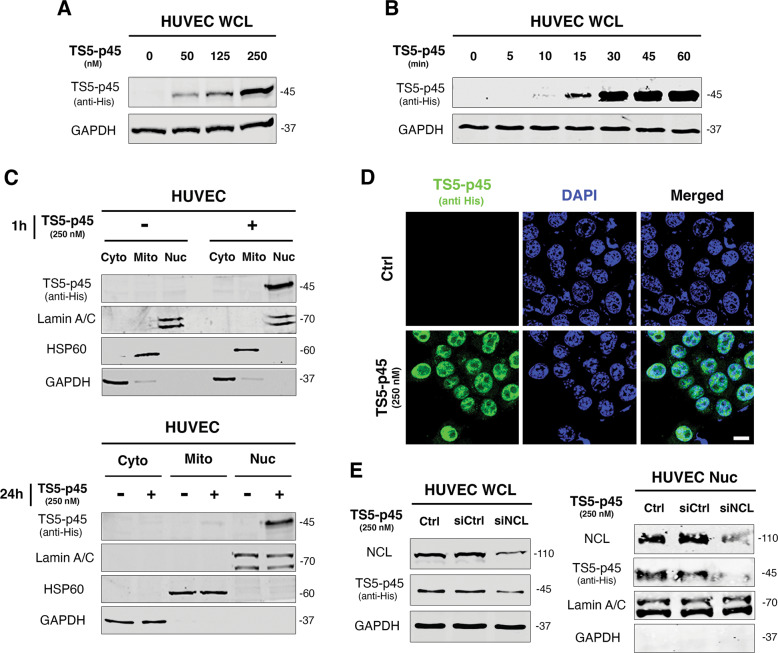
TS5-p45 is internalized and trafficked to the nucleus upon interacting with cell surface NCL. **A**, **B** Dose- and time-dependent TS5-p45 internalization into HUVECs; **A** various concentrations and **B** various lengths of time, GAPDH was used as a loading control. **C**, **D** TS5-p45 was predominantly translocated into the nucleus. **C** HUVECs treated with TS5-p45 for 1 h and 24 h was fractionated, and TS5-p45 was shown by anti-His blotting. Lamin A/C (Nuc), GAPDH (Cyto), and HSP60 (Mito) were used as loading controls for respective fractions. **D** HUVECs treated with TS5-p45 were stained by anti-His (TS5-p45, green) and DAPI (Nuc marker, blue). Scale bar represents 200 μm. **E** siRNA knockdown of NCL reduced the internalization and nuclear translocation of TS5-p45. siNCL-transfected HUVECs were treated with TS5-p45 and fractionated into WCL and Nuc fractions. Endogenous NCL levels in WCL and Nuc fractions were analyzed by anti-NCL blotting. GAPDH (Cyto) and Lamin A/C (Nuc) were used as loading controls.

Upon interacting with HUVECs, TS5-p45 is internalized into ECs in a dose- and time-dependent manner (Fig. 4A, B), with TS5-p45 internalization first observed within 10 min of interaction. Using cell fractionation, we show that TS5-p45 is trafficked to the nucleus (Nuc) after 1 h of incubation with ECs, with very little of it detected in the cytosol (Cyto) or mitochondria (Mito). At 24 h post-treatment, TS5-p45 remained in the nucleus (Fig. 4C). The purity of the subcellular fractions was confirmed by using various markers, including Lamin A/C for Nuc, HSP60 for Mito, and GAPDH for Cyto (Fig. 4C). Immunofluorescence staining further confirmed the efficient nuclear trafficking of TS5-p45 after 1 h of interaction with ECs (Fig. 4D).

To reveal if the internalization and nuclear trafficking of TS5-p45 are dependent on cell surface NCL, we knocked down NCL with siRNA and incubated siNCL-transfected HUVECs with TS5-p45 for 1 h. As shown in Fig. 4E, upon knockdown of NCL, TS5-p45 internalization and nuclear trafficking are both significantly reduced. These results indicate that cell surface NCL mediates the internalization and nuclear trafficking of extracellular TS5-p45.

### Intact late endosomes (LEs) are required for nuclear trafficking and pro-apoptotic activity of TS5-p45

**Fig. 5:**
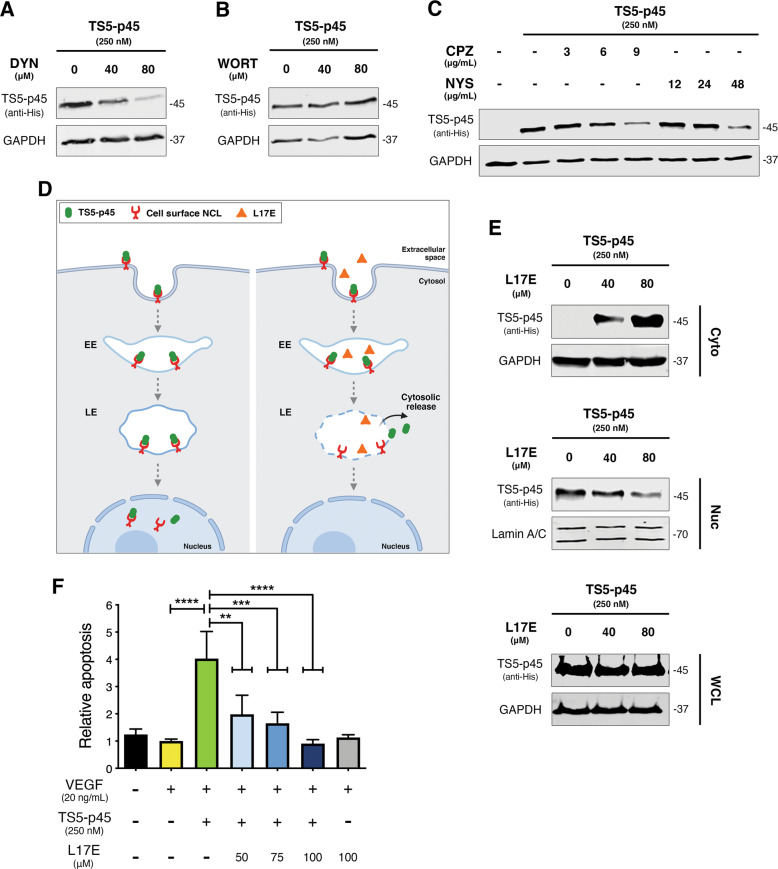
Intact LEs are required for nuclear trafficking and pro-apoptotic activity of TS5-p45. **A–C** TS5-p45 was internalized into HUVECs through clathrin- and caveolin-dependent endocytosis. HUVECs pre-treated with **A** DYN, **B** WORT, and **C** CPZ and NYS, respectively, before being incubated with TS5-p45. TS5-p45 internalization is shown by anti-His blotting. **D** A schematic illustration of the action of the endosomolytic L17E peptide, which disrupts the LEs and leads to the release of endosomal cargo into the cytosol. EE, early endosome; LE, late endosome (Created with BioRender.com). **E** L17E suppresses TS5-p45 translocation to the nucleus. HUVECs were pre-treated with L17E peptide followed by incubation with TS5-p45. TS5-p45 presence in Cyto, Nuc, and WCL fractions was detected by anti-His WB. Loading controls used are GAPDH (Cyto and WCL) and Lamin A/C (Nuc). **F** L17E quenched the pro-apoptotic activity of TS5-p45. HUVECs pre-treated with L17E peptide before TS5-p45 treatment to induce apoptosis. Apoptosis measurements were done at 24 h post-treatment and normalized to VEGF-containing condition. Data shown are mean ± SD from three independent experiments in triplicates. Statistical analysis was performed by one-way ANOVA. **p < 0.01, ***p < 0.001, and ****p < 0.0001.

To understand the significance of the internalization for the pro-apoptotic activity of TS5-p45, we inhibited various endocytosis pathways, including clathrin-dependent, caveolin-dependent, and macropinocytosis. Clathrin- and caveolin-dependent pathways comprise dynamin protein in coating assemblies, while macropinocytosis, driven by actin polymerization, does not require coating assembly [39, 40]. DYN blocks clathrin- and caveolin-dependent (dynamin-dependent) pathways [41, 42], while WORT blocks macropinocytosis [43]. DYN dose-dependently hindered TS5-p45 internalization (Fig. 5A), whereas WORT did not affect TS5-p45 internalization (Fig. 5B), suggesting that TS5-p45 internalization is via a dynamin-dependent pathway. Subsequently, CPZ and NYS were used to block clathrin- and caveolin-dependent pathways, respectively. Both CPZ and NYS dose-dependently hindered TS5-p45 internalization (Fig. 5C), suggesting that both clathrin- and caveolin-dependent endocytosis pathways are involved in mediating TS5-p45 internalization.

We then investigated the role of the endosomal protein trafficking pathway using an endosomolytic peptide L17E, a cationic amphiphilic peptide with specific membrane lytic activity for LEs. Adding L17E into cell culture media will lead to this peptide being endocytosed into cells and trafficked to LEs. Within the acidic environment of LEs, this peptide perturbs and lyses the LE membrane, leading to disruption of LE membrane and release of LE content to the cytosol as depicted in Fig. 5D [32]. As shown in Fig. 5E, L17E peptide dose-dependently increased TS5-p45 in the cytosol, with a concomitant decrease of nuclear-localized TS5-p45 without changing the total amount of internalized TS5-p45. These results suggest that LEs play an essential role in the nuclear trafficking of TS5-p45.

To check if LE-mediated nuclear trafficking of TS5-p45 is important for its pro-apoptotic activity, we examined the impact of L17E on TS5-p45-induced apoptosis. As shown in Fig. 5F, L17E diminished the TS5-p45 induced apoptosis in a dose-dependent manner. These results together indicate that LEs are necessary for the nuclear trafficking and pro-apoptotic function of TS5-p45.

### Cell surface NCL shuttles extracellular TS5-p45 to the nucleus

**Fig. 6:**
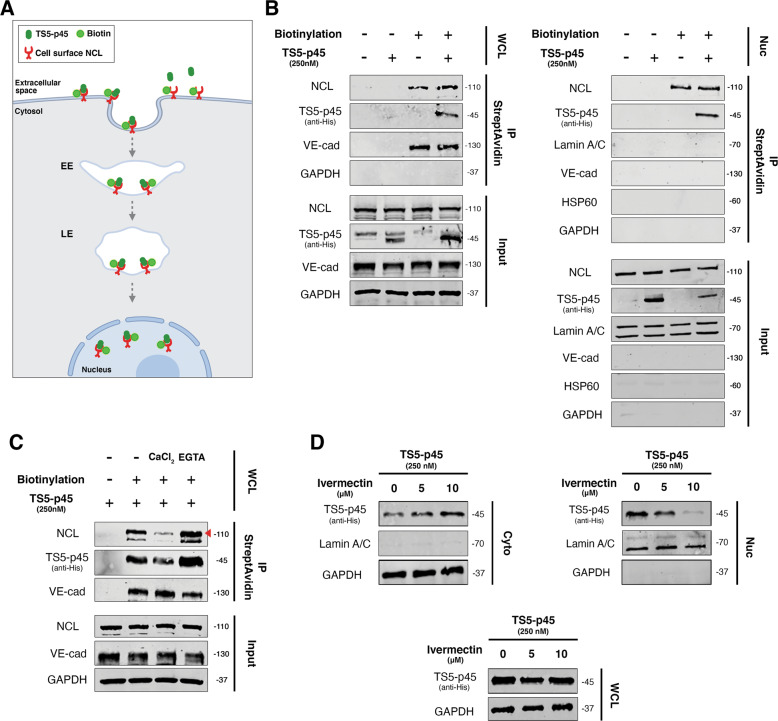
Cell surface NCL shuttles extracellular TS5-p45 to the nucleus. **A** A schematic illustration depicts the biotinylation strategy used to investigate nuclear shuttling of surface NCL and its ligand TS5-p45. Biotinylation of intact HUVECs was performed to biotinylate cell surface NCL, and the biotinylated NCL was then tracked upon TS5-p45 interaction. EE early endosome; LE late endosome (Created with BioRender.com). **B** Cell surface NCL mediates the internalization and nuclear trafficking of TS5-p45. Biotinylated HUVECs treated with TS5-p45 were fractionated, and biotinylated proteins with their binding partners were precipitated by streptavidin for subsequent WB detection. Markers for cell surface (VE-cadherin) and Cyto and WCL (GAPDH) samples were used. Markers for various cell fractions were also used as described in other figures. **C** Amount of TS5-p45 internalization correlated with cell surface NCL expression level. HUVECs pre-treated with CaCl_2_ and EGTA respectively were subjected to biotinylation and TS5-p45 treatment. Biotinylated proteins and their binding partners in WCL were precipitated by streptavidin. **D** Inhibiting importin α1/ß1 suppressed the nuclear trafficking of TS5-p45. HUVECs were pre-treated with Ivermectin before being treated with TS5-p45. Intracellular trafficking was detected by cell fractionation and WB.

Cell surface NCL has been reported to shuttle various extracellular ligands into different subcellular localizations, including the nucleus [44–46]. For instance, cell surface NCL shuttles extracellular endostatin to the EC nucleus [45]. To check if NCL also shuttles extracellular TS5-p45 to the nucleus, we labeled cell surface proteins by biotinylation. As depicted in the schematic diagram in Fig. 6A, cell surface biotinylated HUVECs were treated with TS5-p45 to allow TS5-p45 to be endocytosed and trafficked to the nucleus before harvesting for fractionation. After streptavidin pulldown, the precipitated lysates were probed with NCL and TS5-p45 by WB. Biotinylated cell surface NCL was precipitated down by streptavidin from both WCL and Nuc fractions (Fig. 6B) along with its binding partner TS5-p45. The integrity of cell surface biotinylation was demonstrated by the streptavidin precipitation of plasma membrane VE-cadherin, but not cytosolic GAPDH from WCL (Fig. 6B, left panel) and Lamin A/C from the Nuc fraction (Fig. 6B, right panel). The purity of the Nuc fraction was demonstrated by the presence of Lamin A/C, but not VE-cadherin (plasma membrane), GAPDH (Cyto), and HSP60 (Mito). Thus, the biotinylated NCL detected in WCL, and Nuc fraction can only come from the external surface of the plasma membrane.

It has been reported that cell surface NCL mediates the internalization of extracellular ligands in a calcium-dependent manner [23]. We, therefore, modified the calcium concentration in the EC environment using CaCl_2_ and EGTA pre-treatments and examined its impact on NCL-mediated extracellular TS5-p45 shuttling to the nucleus. Indeed, CaCl_2_ treatment led to a decrease in biotinylated cell surface NCL with a concomitant reduction in cell surface NCL-bound TS5-p45 (Fig. 6C). In contrast, EGTA treatment led to an accumulation/increase in biotinylated cell surface NCL with a concomitant increase in cell surface NCL-bound TS5-p45 (Fig. 6C). Under the same CaCl_2_ and EGTA treatment conditions, no change in cell surface VE-cadherin level and total NCL was observed. These findings further support the notion that cell surface NCL mediates the internalization and nuclear trafficking of extracellular TS5-p45.

NCL contains a bipartite nuclear localization signal (NLS), and importins are known to recognize the NLS of NCL to mediate the nuclear import of NCL and its extracellular ligand as a complex [45, 47]. Therefore, we examined the role of importins in the nuclear trafficking of the NCL-TS5-p45 complex using an importin inhibitor Ivermectin. Ivermectin dose-dependently enhanced the cytosolic level of TS5-p45 with concomitant suppression of nuclear-localized TS5-p45 while the total internalized intracellular TS5-p45 remained constant (Fig. 6D). This result suggests that importin may be involved in the trafficking of the NCL-TS5-p45 complex to the nucleus.

### TS5-p45 alters the expression of apoptosis-related genes in ECs

**Fig. 7:**
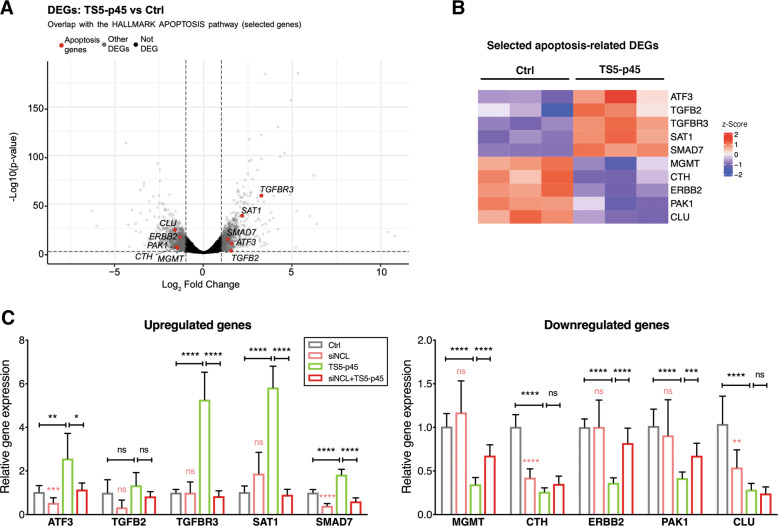
TS5-p45 alters the expression of apoptosis-related genes in ECs. **A** RNA-seq revealed DEGs, shown as downregulated genes (log_2_foldchange < −1) and upregulated genes (log_2_foldchange > 1) with adjusted *p*-value < 0.05, for TS5-p45 vs Ctrl. DEGs with and without significance were shown in gray and black, respectively, while selected 10 apoptosis-related DEGs with significance were highlighted in red. **B** Gene expression changes of the selected 10 apoptosis-related genes were visualized with heatmap across Ctrl and TS5-p45 samples in triplicates. **C** qRT-PCR validation experiments for upregulated genes; *ATF3*, *TGFB2*, *TGFBR3*, *SAT1*, *SMAD7*, and downregulated genes; *MGMT*, *CTH*, *ERBB2*, *PAK1*, and *CLU*. siNCL-transfected ECs were included for TS5-p45 treatment. Gene expression levels under TS5-p45 and siNCL+TS5-p45 treatments were normalized to those of Ctrl treatment and shown as fold change. Data shown are mean ± SD from three independent experiments in triplicates. Statistical analysis was performed by two-way ANOVA. ns, not significant; *p < 0.05; **p < 0.01; ***p < 0.001; ****p < 0.0001.

To further unravel the cellular mechanisms contributing to TS5-p45-induced EC apoptosis, we investigated the altered transcriptional landscape in apoptotic ECs under TS5-p45 treatment via RNA-seq. In total, there are 1054 downregulated and 953 upregulated protein-coding genes whose mRNA levels have changed more than two-fold at 12 h post-TS5-p45 treatment (log_2_ fold change < −1 or > 1 and adjusted *p-*value < 0.05, Table S2). Overlapping these DEGs with the Hallmark apoptosis gene set identified 26 apoptosis-related genes (Table S3 and Fig. 7A). We validated 10 of these apoptosis-related DEGs by qRT-PCR, including 5 apoptosis-inducing genes that are upregulated; *ATF3*, *TGFB2*, *TGFBR3*, *SAT1*, *SMAD7*, and 5 apoptosis-suppressing genes that are downregulated; *MGMT*, *CTH*, *ERBB2*, *PAK1*, and *CLU* (Fig. 7B). To determine if these 10 apoptosis-related genes are important for TS5-p45-induced apoptosis, we knocked down NCL in ECs using siNCL transfection and examined if NCL knockdown would alleviate TS5-p45-induced expression changes. As shown in Fig. 7C, while siNCL knockdown alone did not change the mRNA expression of 5 of the 10 validated genes (*TGFBR3*, *SAR1*, *MGMT*, *ERBB2*, and *PAK1*), siNCL transfection rescued TS5-p45-induced alterations of their expressions, including suppressing the upregulation of *TGFBR3* and *SAT1* while restoring the downregulation of *MGMT*, *ERBB2*, and *PAK1*. Together with the findings that siNCL transfection suppressed TS5-p45-induced apoptosis, these 5 genes potentially contribute to TS5-p45-NCL-mediated apoptosis. For the other 5 genes, the expression of 4 of them (*ATF3*, *SMAD7*, *CTH*, and *CLU*) are already significantly downregulated by siNCL transfection alone, making it difficult to conclude if they are important in TS5-p45-mediated gene expression alteration and apoptosis. No significant effect is observed for the last gene, *TGFB2*, by siNCL transfection alone or TS5-p45 treatment. Notably, TS5-p45 treatment of ECs did not change NCL expression at protein or mRNA levels (Fig. S8). These results together suggest that TS5-p45 treatment leads to expression changes of many genes in ECs, and a large portion of them in an NCL-dependent manner, suggesting their potential importance in TS5-p45-induced EC apoptosis.

## Discussion

**Fig. 8:**
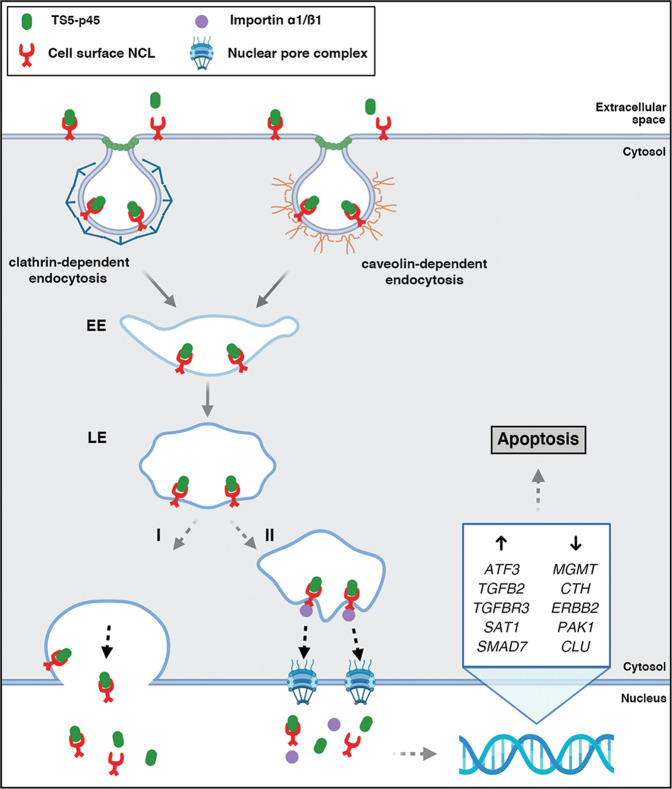
A schematic illustration of the pro-apoptotic mechanism of action of TS5-p45 in ECs. Upon binding to its cell surface receptor NCL, TS5-p45 gets internalized through clathrin- and caveolin-dependent endocytosis and trafficked to LEs. Thereafter, two possible mechanisms may be involved in the nuclear trafficking of TS5-p45. **I** LEs shuttle the NCL-TS5-p45 complex to the nucleus via fusion with the nuclear envelope. **II** NCL-TS5-p45 complex is transported to the nucleus by a process involving importin α1/ß1. TS5-p45-treated ECs exhibits upregulation of caspase-dependent apoptosis-inducing genes; *ATF3*, *TGFB2*, *TGFBR3*, *SAT1*, *SMAD7*, and downregulation of caspase-dependent apoptosis-suppressing genes; *MGMT*, *CTH*, *ERBB2*, *PAK1*, and *CLU*. Finally, nuclear-translocated TS5-p45 activates upstream targets of Cas-8 (extrinsic) and Cas-9 (intrinsic)-dependent apoptosis pathways, resulting in Cas-3 activation and apoptosis. EE early endosome; LE late endosome (Created with BioRender.com).

As summarized in Fig. 8, this work identified cell surface NCL as a novel high-affinity EC surface receptor for the extracellular metalloproteinase ADAMTS5, mediating its pro-apoptotic activity in ECs. We show that cell surface NCL mediates a rapid internalization of TS5-p45, and the TS5-p45-NCL complex is trafficked to the nucleus via LE- and importin-dependent mechanisms. We further demonstrate that the nuclear trafficking of TS5-p45 is essential for its pro-apoptotic activity in ECs. These findings reveal novel biological insights into how ADAMTS5 inhibits angiogenesis.

Cell surface NCL is known to have dual functions in angiogenesis and cancer, depending on the ligand. Multiple ligands with pro-cancer/pro-angiogenesis functions and anti-cancer/anti-angiogenic functions have been identified [48]. Cell surface NCL is known to be preferentially expressed on tumor cells and tumor ECs, and VEGF enhances the cell surface translocation of nucleolar NCL in the tumor microenvironment [21, 48]. The known anti-cancer/anti-angiogenic NCL ligands include endostatin, pseudopeptides HB-19, and N6L, as well as peptide LZ1 [25, 28, 30, 49, 50]. Here, we add ADAMTS5 as a novel anti-cancer/anti-angiogenic ligand of cell surface NCL, inhibiting angiogenesis by triggering EC apoptosis.

Previous reports have shown that extracellular ligands can be internalized and shuttled to the nucleus via cell surface NCL. Examples include endostatin [45], lactoferrin (iron-binding glycoprotein) [44], scuPA (single-chain urokinase-type plasminogen activator) [46], PLA_2_s (Phospholipases A_2_, a major component of snake venoms) [51], and pleiotrophin (heparin-binding growth factor) [52]. Although no detailed trafficking mechanisms have been reported for these extracellular ligands, they indicate the importance of cell surface NCL as a shuttling receptor in mediating the nuclear translocation of extracellular ligands to execute their respective biological activities. In addition, NCL has been previously reported as a potential cell surface receptor for ADAMTS2, another anti-angiogenic member of the ADAMTS family [53]. Our work here adds ADAMTS5 as a new extracellular ligand that is shuttled to the nucleus by cell surface NCL to execute apoptosis in ECs, inhibiting angiogenesis. It has been reported that cell surface NCL shuttles extracellular ligands into the intracellular environment via an active transport mechanism and in a calcium-dependent manner [23]. We showed here that LEs are necessary for the nuclear trafficking of TS5-p45 and its pro-apoptotic function in ECs (Fig. 5E, F). How LEs transport the TS5-p45-NCL complex to the nucleus is still unclear and intriguing. Previously, a bacterial toxin Pseudomonas exotoxin A (PE) was reported to translocate from the extracellular environment to the nucleus within 1 h through nuclear envelope-associated endosomes (NAE), with NAEs undergoing fusion with nuclear envelope to discharge their cargo into the nucleoplasm [54]. Interestingly, no interaction between PE and importin β1 was detected in this study [54]. In another study, a novel endosomal pathway called nuclear envelope invagination-associated late endosomes (N-ALE) was reported to translocate the contents of cancer cell-derived extracellular vesicle (EV) to the nucleus [55]. In this case, subdomains of Rab7^+^ LEs and nuclear envelope invaginations merge to form a sub-nuclear compartment to deliver EV content. Notably, blocking nuclear translocation machinery using an importin β1 inhibitor hinders the nuclear translocation of EV content, suggesting that cargo has to go through the nuclear pore complex [55]. Here, we show that the nuclear trafficking of TS5-p45 via cell surface NCL involves both LEs and importins. TS5-p45 lacks any canonical NLS and is also unlikely to pass through the nuclear pore complex due to its size. Meanwhile, NCL has a bipartite NLS, and TS5-p45 is more likely translocated to the nucleus through recognizing NLS of NCL by importins (Fig. 6D). Incidentally, inhibiting importins was also previously reported to hinder the nuclear trafficking of endostatin by NCL [45].

TS5-p45 is translocated to the nucleus within 1 h and remains in the nucleus for at least 24 h (Fig. 4C), while TS5-p45-induced apoptosis can be first detected after 8 h post-treatment (Fig. S1B). RNA-seq analysis of ECs at 12 h post-TS5-p45 treatment revealed that several apoptosis-inducing genes are upregulated (*ATF3*, *TGFB2*, *TGFBR3*, *SAT1*, and *SMAD7*), while quite a few apoptosis-suppressing genes are downregulated (*MGMT*, *CTH*, *ERBB2*, *PAK1*, *CLU*). These genes have all been previously reported to be involved in caspase-dependent apoptosis in various cell types [56–65]. Furthermore, expression alterations of 5 of the 10 genes validated by qRT-PCR (*TGFBR3*, *SAT1*, *MGMT*, *ERBB2*, *PAK1*) were NCL-dependent, with NCL knockdown alleviated their expression changes under TS5-p45 treatment (Fig. 7C). These results suggest that a large portion of the apoptosis-related genes are involved in TS5-p45-NCL mediated apoptosis. Nevertheless, future studies are required to ascertain if and how they contribute to TS5-p45-induced apoptosis. Targeting cell surface NCL has become a promising therapeutic approach for anti-cancer therapy [66–68]. In conjunction with our previous report that TS5-p45 potently suppressed subcutaneous B16 melanoma growth in mice, our findings here signify that TS5-p45 could be further exploited as a cell surface NCL-targeted anti-cancer therapeutics in the future.

### Reporting summary

Further information on experimental design is available in the Nature Research Reporting Summary linked to this paper.

## Supplementary information

Supplementary Figures S1-S8.

Supplementary Table S1.

Supplementary Table S2.

Supplementary Table S3.

Reporting summary

Author contribution form

Authorship change agreement

## Data Availability

All data for the RNA-seq analysis are available upon request.

## Materials availability

All materials are available upon request.

## Code

The custom R code for the RNA-seq analysis is available upon request.
