# Supplementary material for: Cell surface nucleolin is a novel ADAMTS5 receptor mediating endothelial cell apoptosis
Source: Cell Death Dis. 2022 Feb 23;13(2):172. doi: 10.1038/s41419-022-04618-x (PMC8866485; doi:10.1038/s41419-022-04618-x)
Supplement: Supplementary file 1 — Supplementary Figures S1-S8. [file 41419_2022_4618_MOESM1_ESM.docx]

**Supplementary Figures**


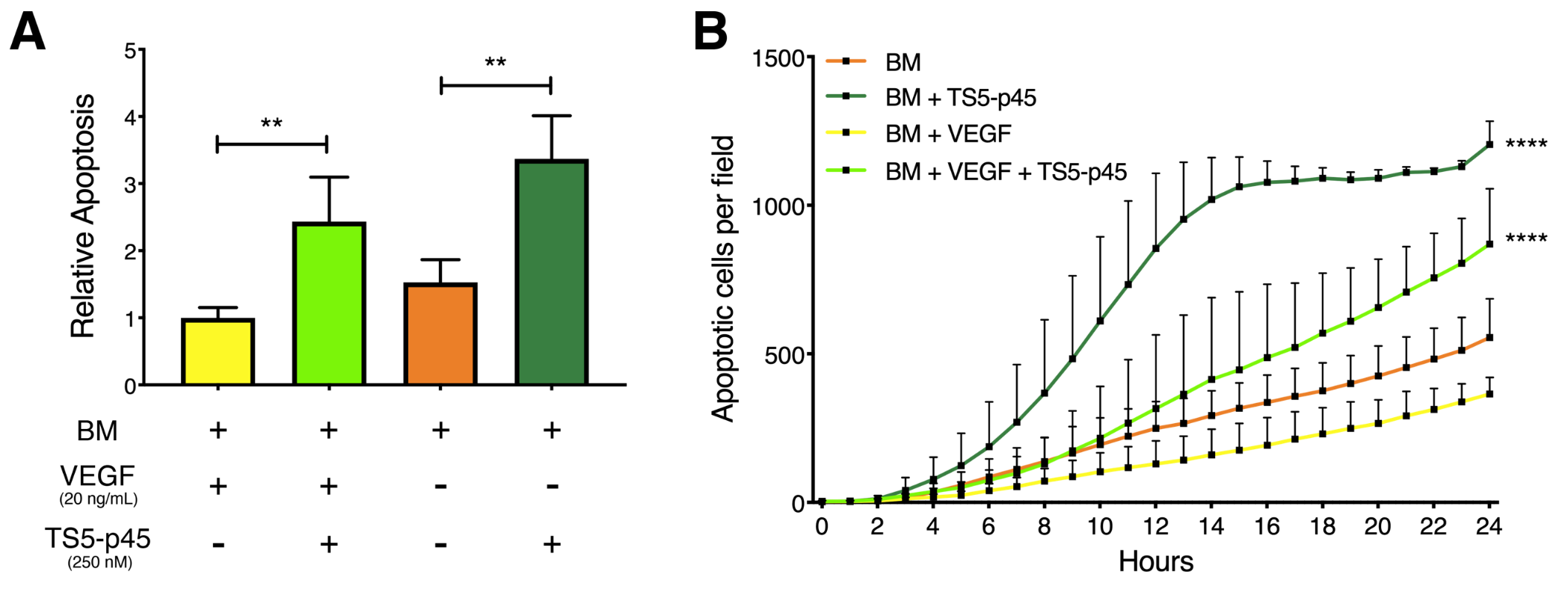


**Fig. S1** TS5-p45-induced EC apoptotic activity in different culture media and time. **A** Apoptotic activities in TS5-p45-treated ECs in the presence and absence of VEGF at 24 h post treatment. BM – Basal Media. **B** Dynamic measurement of EC apoptosis in the first 24 h of TS5-p45 treatment. Control conditions without TS5-p45 treatment were included. Data shown are mean ± SD from three independent experiments in triplicates. Statistical analysis was performed by one-way ANOVA **(A)** and two-way ANOVA **(B)**, respectively. ***p* < 0.001, *****p* < 0.0001.

**_
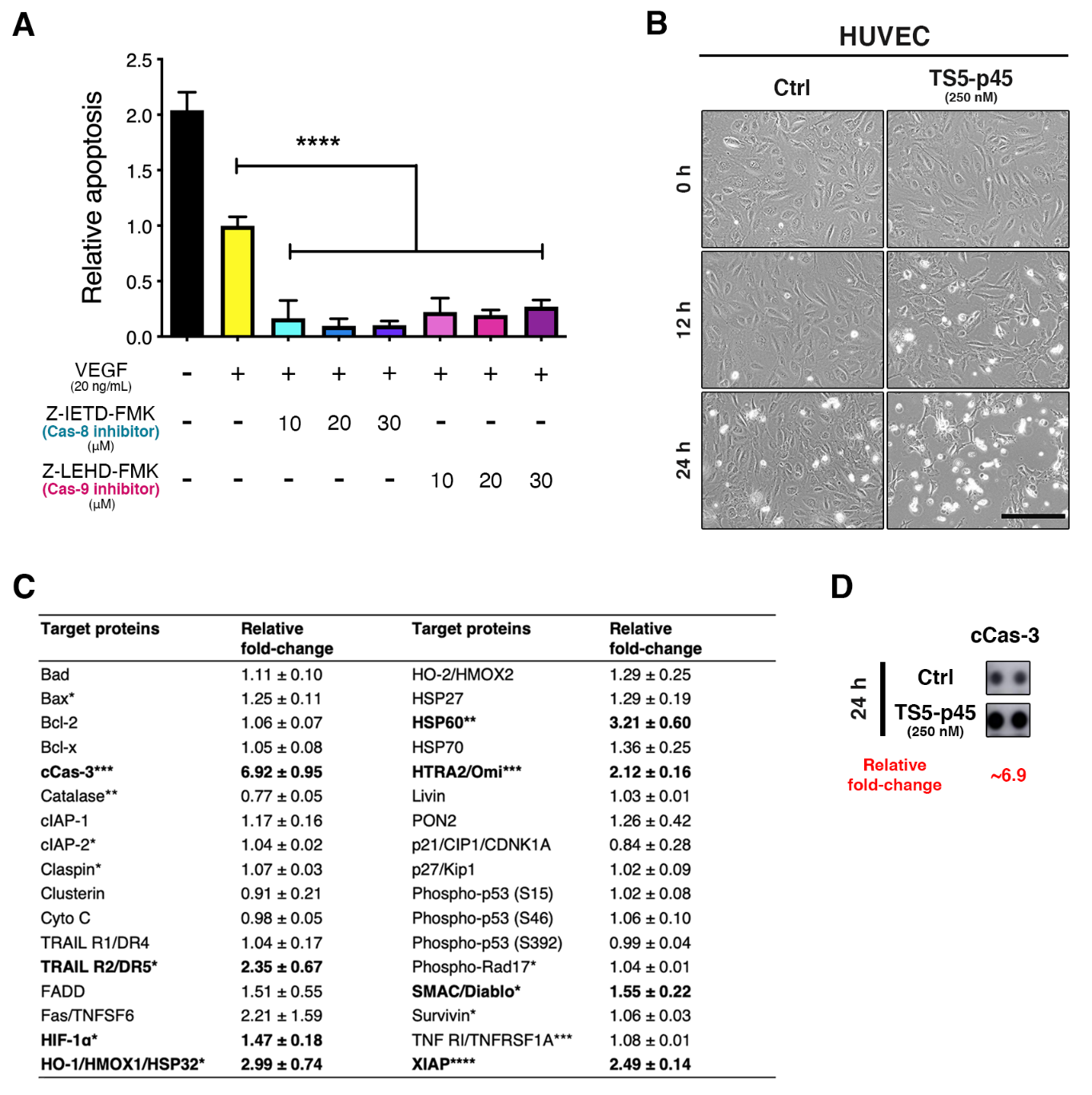
_**

**Fig. S2 A** Cas-8 and Cas-9 inhibitors alone did not induce any cytotoxic effect in HUVECs. Apoptosis was measured at 24 h post-treatment. Relative apoptosis normalized to the VEGF-containing condition is presented. Data shown are mean ± SD from three independent experiments in triplicates. Statistical analysis was performed by one-way ANOVA. *****p* < 0.0001. **B** Phase-contrast light microscopy images of TS5-p45-treated HUVECs showing apoptosis-related morphological changes. Scale bar represents 200 µm. **C** Apoptosis-related pathway proteins upregulated at 24 h post-TS5-p45 treatment. Proteome profiler human apoptosis array was probed using TS5-p45-treated HUVEC lysates. Relative fold-changes (TS5-p45/Ctrl) for each protein were shown. Significantly changed proteins with fold-change ≥ 1.5 were shown in bold. Data shown are mean ± SD from three independent experiments in duplicates. Statistical analysis was performed by Student’s t-test. **p* < 0.05; ***p* < 0.01; ****p* < 0.001 and *****p* < 0.0001. **D** Representative array shows a drastic increase in cleaved caspase-3 (cCas-3) expression level in TS5-p45-treated HUVECs with a relative fold-change of ~6.9.


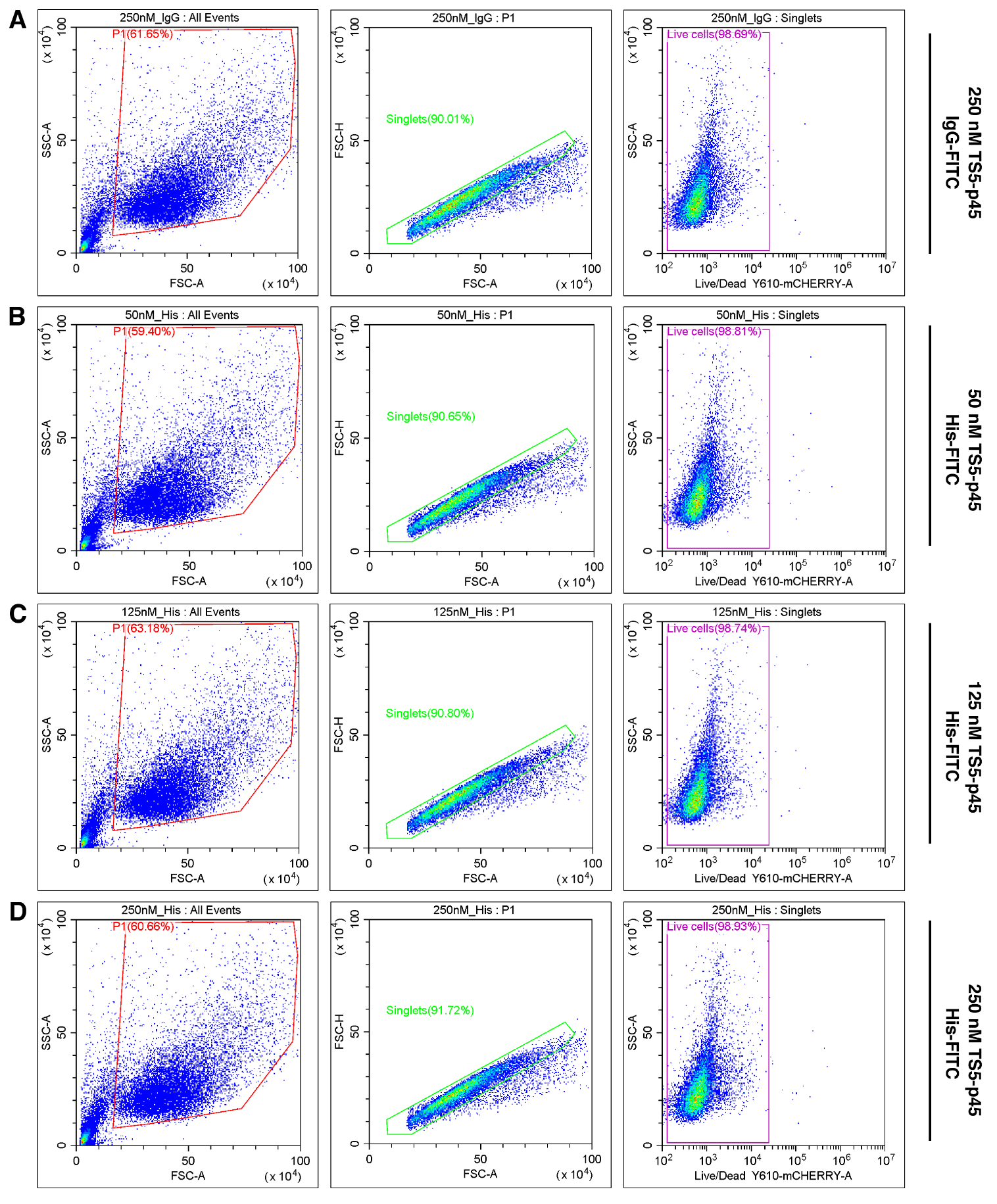


**Fig. S3** Gating settings for flow cytometry analyses of TS5-p45 binding to HUVECs surface. Forward scatter (FSC) versus side scatter (SSC) (light scatter gating) was used to assess the population of cells based on their size and cytoplasmic granularity, respectively, and dead cells and debris were excluded from further analysis (panels on the left column). Pulse (doublet) gating was then used to exclude doublets/clumps, leaving just singlet cells for further analysis (panels on the middle column). Live/dead gating was used to exclude nonviable cells using LIVE/DEAD™ Red Dead Cell Stain (panels on the right column). Gating strategy were applied to all conditions, namely HUVECs with **(A)** 250 nM TS5-p45 + IgG-FITC staining, **(B)** 50 nM TS5-p45 + His-FITC staining, **(C)** 125 nM TS5-p45 + His-FITC staining, and **(D)** 250 nM TS5-p45 + His-FITC staining, respectively.


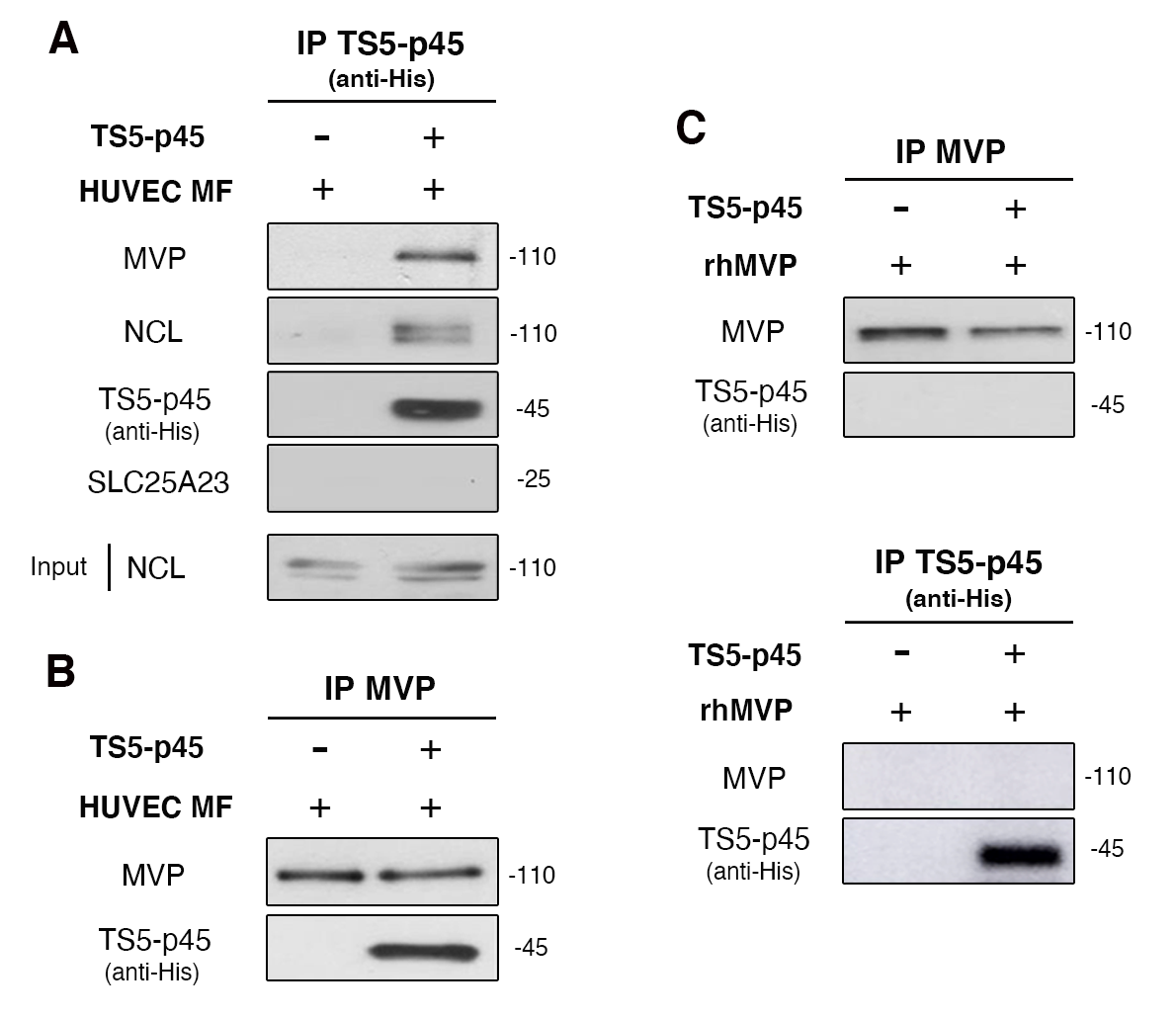


**Fig. S4** SLC25A23 and MVP did not show direct binding to TS5-p45 in co-IP. **A** Binding between TS5-p45 and HUVEC MF-derived NCL, MVP, and SLC25A23 were performed through IP TS5-p45 (anti-His). **B** Binding between TS5-p45 and HUVEC MF-derived MVP was performed through IP MVP. **C** Binding between TS5-p45 and rhMVP was performed through IP MVP and IP TS5-p45 (anti-His).


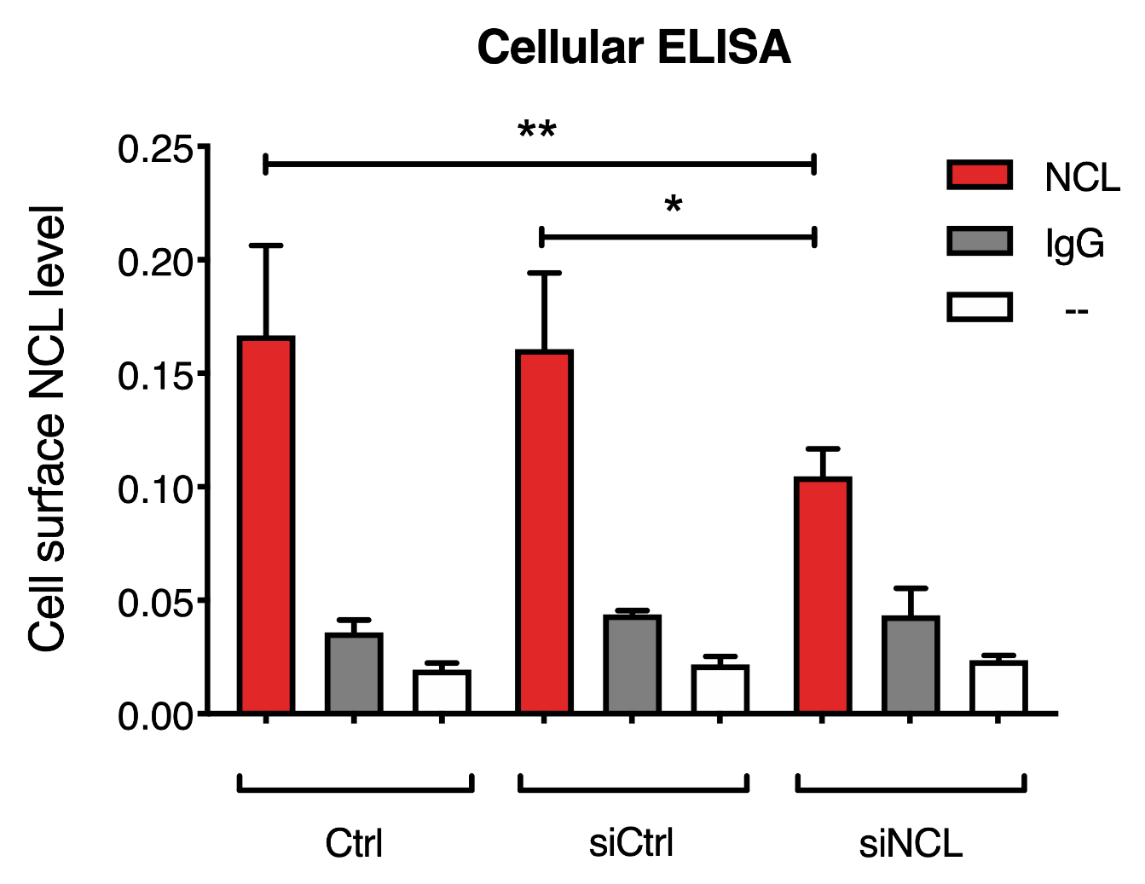


**Fig. S5** siNCL transfection effectively reduced endogenous cell surface NCL expression in HUVECs. Transfected HUVECs were used to measure surface NCL expression level through cellular ELISA by anti-NCL detection. IgG isotype control antibody and no primary antibody control (--) were included as negative controls for all conditions. Data shown are mean ± SD from two independent experiments in triplicates. Statistical analysis was performed by one-way ANOVA. **p* < 0.05; ***p* < 0.01.


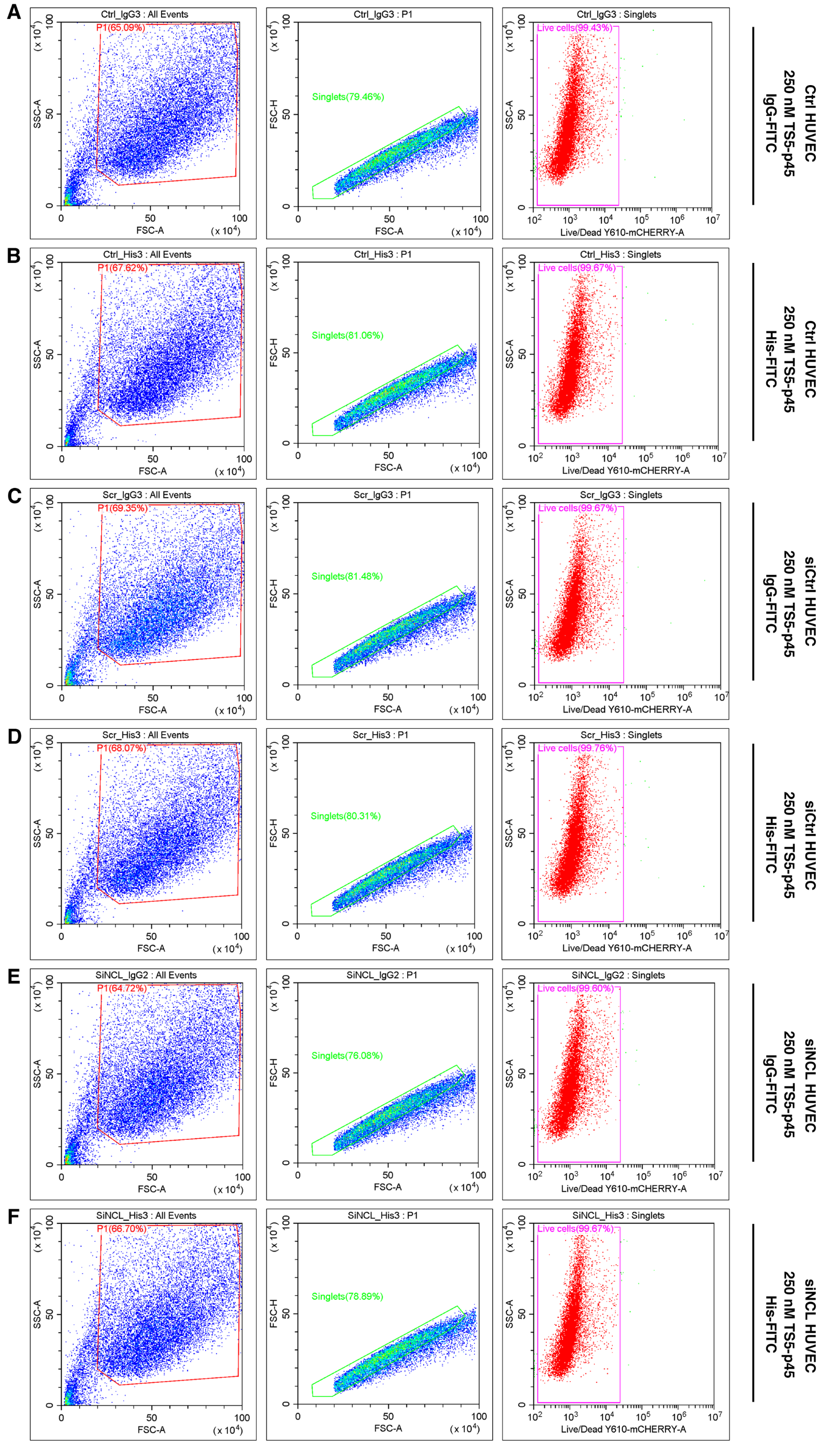


**Fig. S6** Gating settings for flow cytometry analyses of TS5-p45 binding to the surface of transfected HUVECs. Forward scatter (FSC) versus side scatter (SSC) (light scatter gating) was used to assess the population of cells based on their size and cytoplasmic granularity, respectively, and dead cells and debris were excluded from further analysis (panels on the left column). Pulse (doublet) gating was then used to exclude doublets/clumps, leaving just singlet cells for further analysis (panels on the middle column). Finally, live/dead gating was used to exclude nonviable cells using LIVE/DEAD™ Red Dead Cell Stain (panels on the right column). Gating strategy was applied to all conditions, namely **(A)** Ctrl HUVECs with TS5-p45 + IgG-FITC staining, **(B)** Ctrl HUVECs with TS5-p45 + His-FITC staining, **(C)** siCtrl-transfected HUVECs with TS5-p45 + IgG-FITC staining, **(D)** siCtrl-transfected HUVECs with TS5-p45 + His-FITC staining, **(E)** siNCL-transfected HUVECs with TS5-p45 + IgG-FITC staining, and **(F)** siNCL-transfected HUVECs with TS5-p45 + His-FITC staining, respectively

**
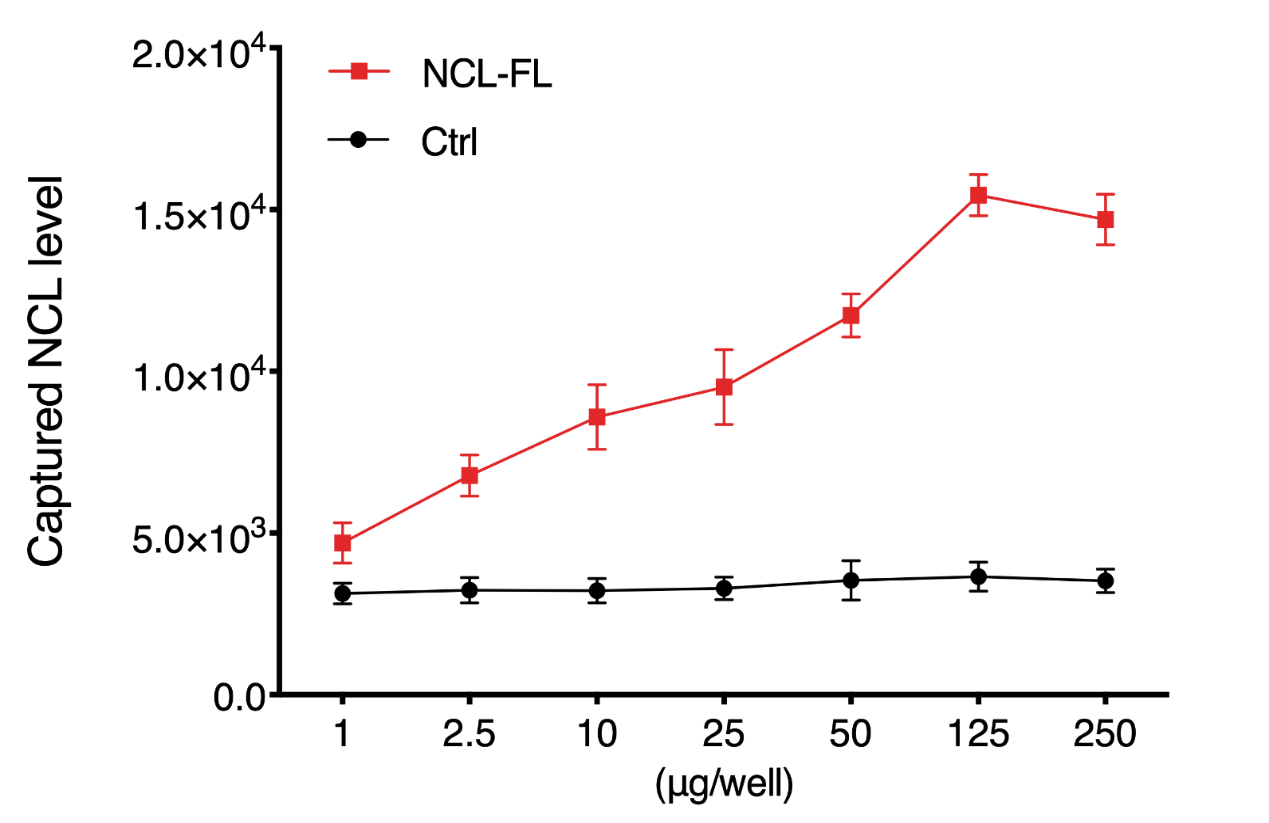
**

**Fig. S7** Specific and saturation binding of FLAG-tagged NCL-FL vs. Ctrl in anti-FLAG antibody-coated 96-well plate. Anti-FLAG antibody-coated plate was treated with different quantities of FLAG-tagged NCL-FL vs. Ctrl lysates. Plate-bound NCL-FL was detected using rabbit anti-NCL and anti-rabbit Alexa Fluor 488. Data shown are mean ± SD from two independent experiments in triplicates.


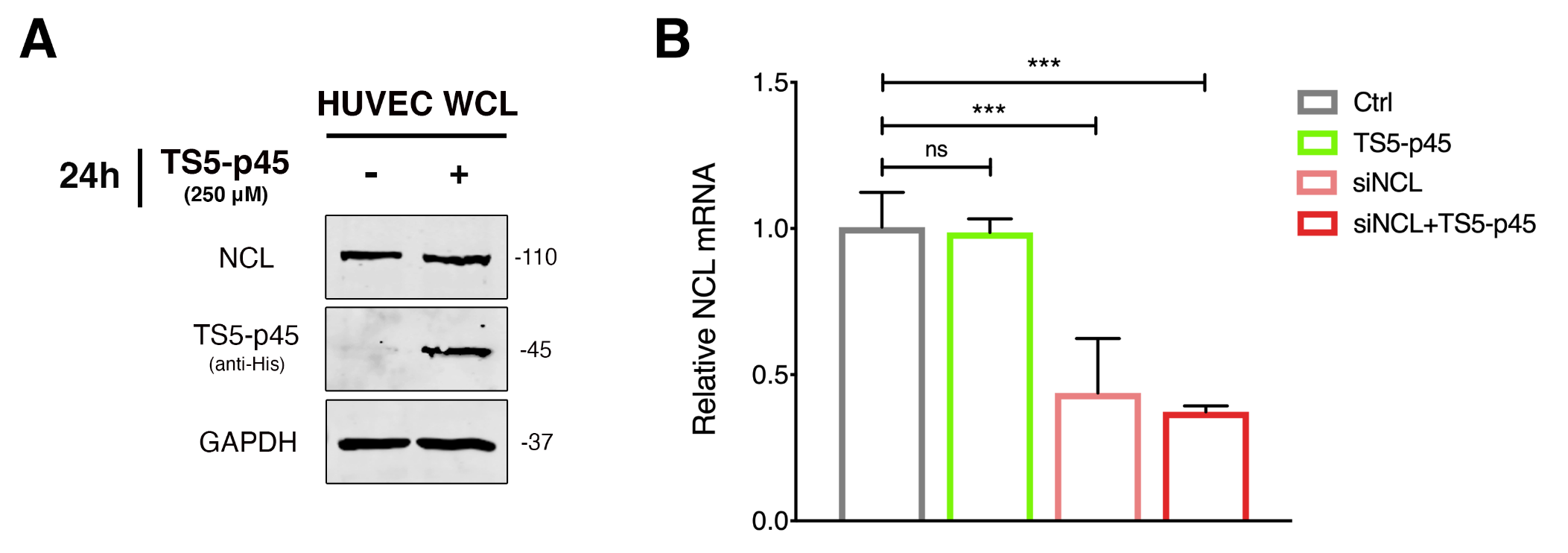


**Fig. S8** NCL expression level in ECs under TS5-p45 treatment. **A** NCL protein level under TS5-p45 treatment analyzed by Western blot. Internalized TS5-p45 level in WCL were analyzed by anti-His blotting. GAPDH was used as a loading control. **B** NCL mRNA level analyzed by qRT-PCR. NCL mRNA levels with TS5-p45, siNCL, and siNCL+TS5-p45 treatments were normalized to those of Ctrl treatment, respectively, and shown as fold change. Data shown are mean ± SD from three independent experiments in triplicates. Statistical analysis was performed by one-way ANOVA. ns, not significant; ****p* < 0.001.
