# Supplementary material for: Cell surface nucleolin is a novel ADAMTS5 receptor mediating endothelial cell apoptosis
Source: Cell Death Dis. 2022 Feb 23;13(2):172. doi: 10.1038/s41419-022-04618-x (PMC8866485; doi:10.1038/s41419-022-04618-x)
Supplement: Supplementary file 6 — Author contribution form [file 41419_2022_4618_MOESM6_ESM.pdf]

DECLARATION OF CONTRIBUTIONS TO ARTICLE

ADMC

Manuscript Number: CDDIS-21-1364RRR

Journal Name: *Cell Death & Disease* (the 'Journal')

Proposed Title of the Contribution:

Cell surface nucleolin is a novel ADAMTS5 receptor mediating endothelial cell apoptosis (the 'Contribution)

Author(s):

Dogan Can Kirman, Bhuvanasundar Renganathan, Wai Kit Chui, Ming Wei Chen, Neslihan Arife Kaya, Ruowen Ge (the 'Authors')

For all *CDDis* articles, each person named as an author in the published version must be able to show he or she has contributed substantially to the article.

Authorship credit should be based on 1) substantial contributions to conception and design, acquisition of data, or analysis and interpretation of data; 2) drafting the article or revising it critically for important intellectual content; and 3) final approval of the version to be published. Authors should meet conditions 1, 2 and 3.

Any person who cannot be shown to have made a substantial contribution to the article cannot be listed as an author in the final version. The name of any person who is deemed to have made a minor contribution can, however, appear in the Acknowledgments section of the article.

Please complete the table below to indicate the contributions of all named authors to the manuscript.

Author Full Name:	Specification of Contribution to the Manuscript:
Dogan Can Kirman (D.C.K.)	carried out the main experiments, prepared the figures, wrote the manuscript
Bhuvanasundar Renganathan (B.R.)	carried out main experiments, wrote the manuscript
Wai Kit Chui (W.K.C.)	performed the pulldown, MS and IP validation experiments for nucleolin identification
Ming Wei Chen (M.W.C.)	helped with the surface plasmon resonance experiment
Neslihan Arife Kaya (N.A.K.)	performed the RNA-seq analysis
Ruowen Ge (R.G.)	conceptualized the research, obtained the funding, supervised the research, wrote the manuscript

Please complete the table below to indicate the contributions of all named authors to the figures.

Figure 1:

B.R. - Fig. 1A, 1B, 1D, 1E
D.C.K. - Fig. 1C, 1D, S1A, S1B

Figure 2:

D.C.K. - Fig. 2A, 2B, 2F-I, 2K, 2L
W.K.C. - Fig. 2C-E
M.W.C. - Fig. 2J
B.R. - S2A
D.C.K. - Fig. S2B-D

Figure 3:

D.C.K. - Fig. 3A-G
W.K.C. - Fig. S3A-C

Figure 4:

B.R. - Fig. 4A-D
D.C.K. - Fig. 4E
D.C.K. - Fig. S4A-D

Figure 5:

B.R. - Fig. 5A-C, 5E-F
D.C.K. - Fig. 5D
D.C.K. - Fig. S5A-F

Figure 6:

D.C.K. - Fig. 6A-D, S6

Figure 7: N.A.K. - Fig. 7A, 7B
D.C.K. - Fig. 7C, S7

Figure 8: D.C.K. - Fig. 8, S8A-B

* Revisions were done by D.C.K.

Signed for and on behalf of the Author(s):	Print Name:	Date:
[signature]	Ruowen Ge	Jan. 11, 2022
