# Supplementary material for: Cell surface nucleolin is a novel ADAMTS5 receptor mediating endothelial cell apoptosis
Source: Cell Death Dis. 2022 Feb 23;13(2):172. doi: 10.1038/s41419-022-04618-x (PMC8866485; doi:10.1038/s41419-022-04618-x)
Supplement: Supplementary file 7 — Authorship change agreement [file 41419_2022_4618_MOESM7_ESM.pdf]

**From:** Dogan Can Kirman
**To:** Ge Ruowen
**Cc:** Wai Kit Chui; Kayaan@gis.a-star.edu.sg; Chen Ming Wei (Dr); Bhuvanasundar Renganathan
**Subject:** Re: CDDIS-21-1364RRR Initial Quality Check
**Date:** Monday, 17 January 2022 12:38:16 PM

---

Dear All,

I agree with the change of authorship where we added Neslihan Kaya as she helped us with the added RNA-seq data in our revised manuscript.

Regards,
Can

---

**From:** Wai Kit Chui <chui_wai_kit@hotmail.com>
**Sent:** Saturday, 15 January 2022 7:49 pm
**To:** Ge Ruowen <dbsgerw@nus.edu.sg>; Dogan Can Kirman <cankirman@u.nus.edu>; Kayaan@gis.a-star.edu.sg <Kayaan@gis.a-star.edu.sg>; Bhuvanasundar Renganathan <r.bhuvanasundar@gmail.com>; Chen Ming Wei (Dr) <mwchen@ntu.edu.sg>
**Subject:** Re: CDDIS-21-1364RRR Initial Quality Check

- External Email -

Dear All,

I am in agreement with the changes and change of authorship for this manuscript.

Thank you.

Regards,
Wai Kit

---

**From:** Ge Ruowen <dbsgerw@nus.edu.sg>
**Sent:** Friday, January 14, 2022 11:51 AM
**To:** Dogan Can Kirman <cankirman@u.nus.edu>; Kayaan@gis.a-star.edu.sg <Kayaan@gis.a-star.edu.sg>; Bhuvanasundar Renganathan <r.bhuvanasundar@gmail.com>; Chen Ming Wei (Dr) <mwchen@ntu.edu.sg>; Wai Kit Chui <chui_wai_kit@hotmail.com>
**Subject:** FW: CDDIS-21-1364RRR Initial Quality Check

Hi all co-authors,

I am forwarding the below email from Cell Death & Disease editorial office. Although we have previously submitted the authorship change form with everyone's signature, the journal still now request each co-author to email reply that you are agreeable to the change of authorship in order to officially accept our manuscript. Please reply to my email ASAP with your statement that you are agreeable with the change of authorship from the initial submission.

Just to re-state here that in our revised manuscript, we have added Neslihan Arife Kaya as a co-author as we have added RNA-seq data which she helped to analyze.

Regards,

Ruowen GE, PhD; Associate Professor, Department of Biological Sciences, National University of Singapore; Block S3, #06-02, 16 Science Drive 4, Singapore, 117558.

---

**From:** cddisease@springernature.com <cddisease@springernature.com>
**Sent:** Thursday, 13 January 2022 11:41 PM
**To:** Ge Ruowen <dbsgerw@nus.edu.sg>
**Subject:** CDDIS-21-1364RRR Initial Quality Check

- External Email -

Dear Professor Ge,

In checking in your manuscript submitted to Cell Death & Disease it has come to our attention that the following must be addressed before we can proceed.

1. Please make sure all tables and figures, including supplementary, are cited in the main text, in numerical order.

2. It has come to our attention that your most recent author list differs from the one in your original submission.

Please request agreement from all authors including additions and deletions, these can be collected in the following way:

Email your co-authors with the change, and ask them to reply to your email confirming that they agree to these changes. Once you have collected these replies, please combine all of the co-authors' email responses in one document and upload this file to your submission.

Your paper is now available for you to edit, you may access via the following link:

https://mts-cddis.nature.com/cgi-bin/main.plex?el=A3CA4UEF2D6LRO1F6A9ftdZqSQtZa5zgqq7gbjyZaTIQZ

(Press/Click on the above link to be automatically sent to the web page.)

Please make the correction(s) as specified above and resubmit your paper following the same steps as before.

If you have any questions please feel free to contact us.

Sincerely,

Editorial Office
Cell Death & Disease
cddisease@springernature.com

This email has been sent through the NPG Manuscript Tracking System NY-610A-NPG&MTS

*Confidentiality Statement:*

*This e-mail is confidential and subject to copyright. Any unauthorised use or disclosure of its contents is prohibited. If you have received this email in error please notify our Manuscript Tracking System Helpdesk team at http://platformsupport.nature.com .*
*Details of the confidentiality and pre-publicity policy may be found here http://www.nature.com/authors/policies/confidentiality.html*
Privacy Policy | Update Profile

Virus-free. www.avg.com
Notice: This email is generated from the account of an NUS alumnus. Contents, views, and opinions therein are solely those of the sender.

**From:** Chen Ming Wei (Dr)
**To:** Ge Ruowen
**Subject:** Re: CDDIS-21-1364RRR Initial Quality Check
**Date:** Monday, 17 January 2022 1:04:57 PM

- External Email -

Hi All,

Yes, I agree with the authorship changes. Thanks!

Best wishes
MW

---

**From:** Ge Ruowen <dbsgerw@nus.edu.sg>
**Sent:** Monday, January 17, 2022 9:52 AM
**To:** Chen Ming Wei (Dr) <mwchen@ntu.edu.sg>
**Subject:** FW: CDDIS-21-1364RRR Initial Quality Check

Hi Ming Wei,

Could you response ASAP? Please use the email reply chain to make things easier.

Ruowen GE, PhD; Associate Professor, Department of Biological Sciences, National University of Singapore; Block S3, #06-02, 16 Science Drive 4, Singapore, 117558.

---

**From:** Ge Ruowen
**Sent:** Friday, 14 January 2022 11:52 AM
**To:** Dogan Can Kirman <cankirman@u.nus.edu>; Kayaan@gis.a-star.edu.sg; Bhuvanasundar Renganathan <r.bhuvanasundar@gmail.com>; Chen Ming Wei (Dr) <mwchen@ntu.edu.sg>; Wai Kit Chui <chui_wai_kit@hotmail.com>
**Subject:** FW: CDDIS-21-1364RRR Initial Quality Check
**Importance:** High

Hi all co-authors,

I am forwarding the below email from Cell Death & Disease editorial office. Although we have previously submitted the authorship change form with everyone's signature, the journal still now request each co-author to email reply that you are agreeable to the change of authorship in order to officially accept our manuscript. Please reply to my email ASAP with your statement that you are agreeable with the change of authorship from the initial submission.

Just to re-state here that in our revised manuscript, we have added Neslihan Arife Kaya as a co-author as we have added RNA-seq data which she helped to analyze.

Regards,

Ruowen GE, PhD; Associate Professor, Department of Biological Sciences, National University of Singapore; Block S3, #06-02, 16 Science Drive 4, Singapore, 117558.

---

**From:** cddisease@springernature.com <cddisease@springernature.com>
**Sent:** Thursday, 13 January 2022 11:41 PM
**To:** Ge Ruowen <dbsgerw@nus.edu.sg>
**Subject:** CDDIS-21-1364RRR Initial Quality Check

- External Email -

Dear Professor Ge,

In checking in your manuscript submitted to Cell Death & Disease it has come to our attention that the following must be addressed before we can proceed.

1. Please make sure all tables and figures, including supplementary, are cited in the main text, in numerical order.

2. It has come to our attention that your most recent author list differs from the one in your original submission.

Please request agreement from all authors including additions and deletions, these can be collected in the following way:

Email your co-authors with the change, and ask them to reply to your email confirming that they agree to these changes. Once you have collected these replies, please combine all of the co-authors' email responses in one document and upload this file to your submission.

Your paper is now available for you to edit, you may access via the following link:

https://mts-cddis.nature.com/cgi-bin/main.plex?el=A3CA4UEF2D6LRO1F6A9ftdZqSQtZa5zgqq7gbjyZaTIQZ

(Press/Click on the above link to be automatically sent to the web page.)

Please make the correction(s) as specified above and resubmit your paper following the same steps as before.

If you have any questions please feel free to contact us.

Sincerely,

Editorial Office
Cell Death & Disease
cddisease@springernature.com

This email has been sent through the NPG Manuscript Tracking System NY-610A-NPG&MTS

*Confidentiality Statement:*

*This e-mail is confidential and subject to copyright. Any unauthorised use or disclosure of its contents is prohibited. If you have received this email in error please notify our Manuscript Tracking System Helpdesk team at http://platformsupport.nature.com .*
*Details of the confidentiality and pre-publicity policy may be found here http://www.nature.com/authors/policies/confidentiality.html*
Privacy Policy | Update Profile

---

CONFIDENTIALITY: This email is intended solely for the person(s) named and may be confidential and/or privileged. If you are not the intended recipient, please delete it, notify us and do not copy, use, or disclose its contents.
Towards a sustainable earth: Print only when necessary. Thank you.

| | |
|---|---|
| **From:** | Bhuvanasundar Renganathan |
| **To:** | Ge Ruowen |
| **Cc:** | Dogan Can Kirman; Kayaan@gis.a-star.edu.sg; Chen Ming Wei (Dr); Wai Kit Chui |
| **Subject:** | Re: FW: CDDIS-21-1364RRR Initial Quality Check |
| **Date:** | Saturday, 15 January 2022 6:02:00 AM |

- External Email -

Dear Prof GE Ruowen and Editor in office CDD,

I have revised the current manuscript and acknowledge change in the authorship, ie addition of Neslihan Arife Kaya as a co-author as she helped in RNA-seq data analysis.

Regards
Bhuvan

On Thu, Jan 13, 2022 at 9:51 PM Ge Ruowen <dbsgerw@nus.edu.sg> wrote:

> Hi all co-authors,
>
> I am forwarding the below email from Cell Death & Disease editorial office. Although we have previously submitted the authorship change form with everyone's signature, the journal still now request each co-author to email reply that you are agreeable to the change of authorship in order to officially accept our manuscript. Please reply to my email ASAP with your statement that you are agreeable with the change of authorship from the initial submission.
>
> Just to re-state here that in our revised manuscript, we have added Neslihan Arife Kaya as a co-author as we have added RNA-seq data which she helped to analyze.
>
> Regards,
>
> Ruowen GE, PhD; Associate Professor, Department of Biological Sciences, National University of Singapore; Block S3, #06-02, 16 Science Drive 4, Singapore, 117558.

**From:** cddisease@springernature.com <cddisease@springernature.com>
**Sent:** Thursday, 13 January 2022 11:41 PM
**To:** Ge Ruowen <dbsgerw@nus.edu.sg>
**Subject:** CDDIS-21-1364RRR Initial Quality Check

- External Email -

Dear Professor Ge,

In checking in your manuscript submitted to Cell Death & Disease it has come to our attention that the following must be addressed before we can proceed.

1. Please make sure all tables and figures, including supplementary, are cited in the main text, in numerical order.

2. It has come to our attention that your most recent author list differs from the one in your original submission.

Please request agreement from all authors including additions and deletions, these can be collected in the following way:

Email your co-authors with the change, and ask them to reply to your email confirming that they agree to these changes. Once you have collected these replies, please combine all of the co-authors' email responses in one document and upload this file to your submission.

Your paper is now available for you to edit, you may access via the following link:

https://mts-cddis.nature.com/cgi-bin/main.plex?el=A3CA4UEF2D6LRO1F6A9ftdZqSQtZa5zgqq7gbjyZaTIQZ

(Press/Click on the above link to be automatically sent to the web page.)

Please make the correction(s) as specified above and resubmit your paper following the same steps as before.

If you have any questions please feel free to contact us.

Sincerely,

Editorial Office
Cell Death & Disease
cddisease@springernature.com

This email has been sent through the NPG Manuscript Tracking System NY-610A-

NPG&MTS

*Confidentiality Statement:*

*This e-mail is confidential and subject to copyright. Any unauthorised use or disclosure of its contents is prohibited. If you have received this email in error please notify our Manuscript Tracking System Helpdesk team at http://platformsupport.nature.com .*

*Details of the confidentiality and pre-publicity policy may be found here http://www.nature.com/authors/policies/confidentiality.html*

Privacy Policy | Update Profile

--
R.Bhuvanasundar

**From:** KAYA Arife Neslihan
**To:** Ge Ruowen; Dogan Can Kirman; Bhuvanasundar Renganathan; Chen Ming Wei (Dr); Wai Kit Chui
**Subject:** Re: CDDIS-21-1364RRR Initial Quality Check
**Date:** Friday, 14 January 2022 1:59:51 PM
**Attachments:** Outlook-sctwogmp.png

---

- External Email -

Dear Prof Ge,

I agree to author changes in the revised manuscript which I was added as a co-author after my help with the added RNA-seq analysis.

Thank you!

**A. Neslihan Kaya, PhD**
Post Doctoral Fellow, Laboratory of Human Genomics
**Genome Institute of Singapore**
60 Biopolis St, Genome, #03-303, Singapore 138672
**E-mail:** kayaan@gis.a-star.edu.sg

Genome Institute of Singapore
GIS

HQastar
ASTARSingapore
ASTARTV
A-STAR

CREATING GROWTH, ENHANCING LIVES

---

**From:** Ge Ruowen <dbsgerw@nus.edu.sg>
**Sent:** 14 January 2022 11:51
**To:** Dogan Can Kirman <cankirman@u.nus.edu>; KAYA Arife Neslihan <kayaan@gis.a-star.edu.sg>; Bhuvanasundar Renganathan <r.bhuvanasundar@gmail.com>; Chen Ming Wei (Dr) <mwchen@ntu.edu.sg>; Wai Kit Chui <chui_wai_kit@hotmail.com>
**Subject:** FW: CDDIS-21-1364RRR Initial Quality Check

Hi all co-authors,

I am forwarding the below email from Cell Death & Disease editorial office. Although we have previously submitted the authorship change form with everyone’s signature, the journal still now request each co-author to email reply that you are agreeable to the change of authorship in order to officially accept our manuscript. Please reply to my email ASAP with your statement that you are agreeable with the change of authorship from the initial submission.

Just to re-state here that in our revised manuscript, we have added Neslihan Arife Kaya as a co-author as we have added RNA-seq data which she helped to analyze.

Regards,

Ruowen GE, PhD; Associate Professor, Department of Biological Sciences, National University of Singapore; Block S3, #06-02, 16 Science Drive 4, Singapore, 117558.

---

**From:** cddisease@springernature.com <cddisease@springernature.com>
**Sent:** Thursday, 13 January 2022 11:41 PM
**To:** Ge Ruowen <dbsgerw@nus.edu.sg>
**Subject:** CDDIS-21-1364RRR Initial Quality Check

- External Email -

Dear Professor Ge,

In checking in your manuscript submitted to Cell Death & Disease it has come to our attention that the following must be addressed before we can proceed.

1. Please make sure all tables and figures, including supplementary, are cited in the main text, in numerical order.

2. It has come to our attention that your most recent author list differs from the one in your original submission.

Please request agreement from all authors including additions and deletions, these can be collected in the following way:

Email your co-authors with the change, and ask them to reply to your email confirming that they agree to these changes. Once you have collected these replies, please combine all of the co-authors' email responses in one document and upload this file to your submission.

Your paper is now available for you to edit, you may access via the following link:

https://mts-cddis.nature.com/cgi-bin/main.plex?el=A3CA4UEF2D6LRO1F6A9ftdZqSQtZa5zgqq7gbjyZaTIQZ

(Press/Click on the above link to be automatically sent to the web page.)

Please make the correction(s) as specified above and resubmit your paper following the same steps as before.

If you have any questions please feel free to contact us.

Sincerely,

Editorial Office
Cell Death & Disease
cddisease@springernature.com

This email has been sent through the NPG Manuscript Tracking System NY-610A-NPG&MTS

*Confidentiality Statement:*

*This e-mail is confidential and subject to copyright. Any unauthorised use or disclosure of its contents is prohibited. If you have received this email in error please notify our Manuscript Tracking System Helpdesk team at http://platformsupport.nature.com .*
*Details of the confidentiality and pre-publicity policy may be found here http://www.nature.com/authors/policies/confidentiality.html*
Privacy Policy | Update Profile
This e-mail and any attachments are only for the use of the intended recipient and may contain material that is confidential, privileged and/or protected by the Official Secrets Act. If you are not the intended recipient, please delete it or notify the sender immediately. Please do not copy or use it for any purpose or disclose the contents to any other person.
